# An Updated Review on Essential Oils from Lauraceae Plants: Chemical Composition and Genetic Characteristics of Biosynthesis

**DOI:** 10.3390/ijms26125690

**Published:** 2025-06-13

**Authors:** Fanglan Wu, Yicun Chen, Ming Gao, Wei Li, Yunxiao Zhao, Yangdong Wang

**Affiliations:** 1 State Key Laboratory of Tree Genetics and Breeding, Chinese Academy of Forestry, Beijing 100091, China; falaner@foxmail.com (F.W.); chenyc@caf.ac.cn (Y.C.); 4862705@163.com (M.G.); weili2015@nefu.edu.cn (W.L.); 2 College of Forest, Nanjing Forestry University, Nanjing 210037, China; 3 Research Institute of Subtropical Forestry, Chinese Academy of Forestry, Hangzhou 311400, China; 4 State Key Laboratory of Tree Genetics and Breeding, Northeast Forestry University, Harbin 150040, China

**Keywords:** useful plant species, oil cell, terpene, biosynthesis, terpene synthases, genome

## Abstract

Globally, plant-derived natural products such as essential oils serve as primary sources of functional substances for spices, pharmaceuticals, and other applications. With the increasing focus on health and well-being, alongside ongoing public health challenges, there is a critical need to enhance the deep utilization of natural plant products. Lauraceae family essential oils, characterized by their aromatic, volatile properties and notable biological activities (e.g., antibacterial, antioxidant, insect-repellent), hold significant application value across fragrance, cosmetics, chemical industries, biological pesticides, and medicine. Integrating multi-disciplinary data from biology, genomics, metabolomics, and related fields can accelerate comprehensive insights into the biosynthesis mechanisms and functional roles of these essential oils, thereby promoting the development and application of Lauraceae natural products. This review systematically summarizes the accumulation patterns and compositional characteristics of essential oils across diverse genera of Lauraceae. It further explores the evolutionary dynamics of terpene synthase (TPS) gene families and key genes involved in terpenoid biosynthesis pathways, leveraging genomic datasets from Lauraceae species. Finally, the review highlights future research trends for optimizing Lauraceae essential oil resource utilization and advancing molecular breeding of high-oil-content species within the family.

## 1. Introduction

Plants generate hundreds of thousands of metabolites that play pivotal roles in growth, development, and resistance to biotic and abiotic stresses, enabling plants to adapt to dynamic environmental changes. Plant-derived natural products not only provide pharmaceuticals but also serve as fundamental structural components for human health [1,2]. Given the significance of plant metabolism for development and adaptation, as well as its substantial economic and health value to human society, multi-level studies incorporating genomics, transcriptomics, proteomics, and metabolomics have been undertaken to elucidate the synthesis of metabolites and the regulatory genes involved.

Lauraceae, a prominent family of woody plants (excluding *Cassytha*, a parasitic twining herb), comprises tree species of significant economic and ecological importance [3]. Lauraceae plants serve as a rich resource of essential oils, primarily due to the exceptionally high oil content in their branches, leaves, and seeds, which contribute substantially to human societal economic and health benefits [3,4,5]. The family exhibits remarkable taxonomic diversity and broad geographic distribution, encompassing approximately 2500 to 3000 species across 50 genera, predominantly distributed in tropical and subtropical regions such as Southeast Asia, the Mediterranean, and Central and South America [3,6,7]. This diversity underpins the rich compositional variety of Lauraceae essential oils and enhances their development and utilization potential. However, the evolutionary history and phylogenetic relationships of Lauraceae plants remain complex. Meanwhile, the distribution patterns and diversity of essential oil content and components within the family are still unclear, and strategies for promoting efficient plant-based essential oil production are incomplete. Collectively, these challenges hinder the development and utilization of Lauraceae plant resources, as well as the improvement of essential oil quality and production efficiency. Therefore, it is crucial to develop strategies that harness the diversity of plant essential oils while improving their quality and efficiency to advance the utilization of Lauraceae essential oils.

In recent years, substantial research has been conducted on the essential oil characteristics of the Lauraceae family; however, a comprehensive synthesis of the distribution patterns and regulatory mechanisms underlying essential oil synthesis remains lacking. This review aims to systematically characterize the essential oil profiles of each genus within Lauraceae and dissect the biosynthetic mechanisms of terpenoids in these plants. To this end, we have critically reviewed studies on the chemical composition of essential oils and terpenoid biosynthesis in Lauraceae species published over the past two decades (2004–2024). Additionally, future research directions are discussed to provide a theoretical foundation for the sustainable development of Lauraceae plant resources and the optimization of essential oil quality and production efficiency.

## 2. Lauraceae Essential Oils and Their Use by Humans

**Figure 1:**
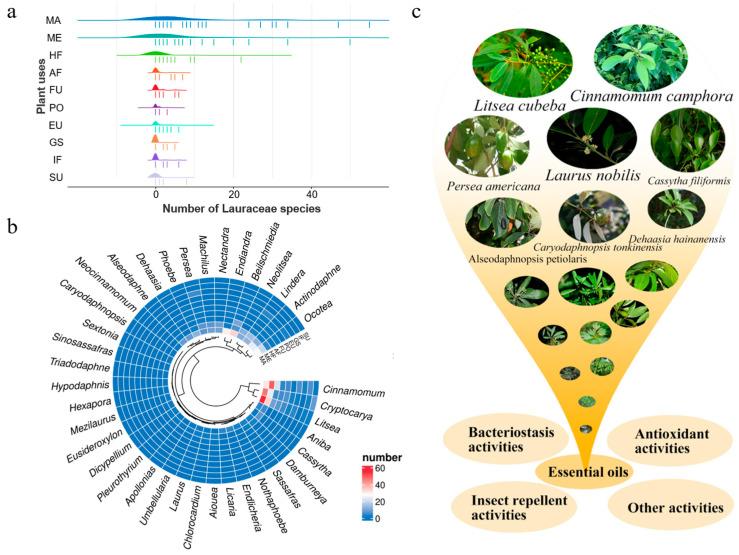
The uses of Lauraceae species. (**a**) The number of Lauraceae species in different plant uses. In the figure, the horizontal coordinate values corresponding to the sticks indicate the number of species in 38 genera (*Actinodaphne*, *Aiouea*, *Alseodaphne*, *Aniba, Apollonias*, *Beilschmiedia*, *Caryodaphnopsis*, *Cassytha*, *Chlorocardium*, *Cinnamomum*, *Cryptocarya*, *Damburneya*, *Dehaasia, Dicypellium*, *Endiandra*, *Endlicheria*, *Eusideroxylon*, *Hexapora*, *Hypodaphnis*, *Laurus*, *Licaria*, *Lindera*, *Litsea*, *Machilus*, *Mezilaurus*, *Nectandra*, *Neocinnamomum*, *Neolitsea*, *Nothaphoebe*, *Ocotea*, *Persea*, *Phoebe*, *Pleurothyrium*, *Sassafras*, *Sextonia*, *Sinosassafras*, *Triadodaphne*, *Umbellularia*) of Lauraceae used for the respective purposes, while the humps represent the enrichment degree. (**b**) Cluster analysis of plant uses in each genus of Lauraceae. The heatmap was generated using the complete-linkage hierarchical clustering algorithm and Euclidean distance metric on unprocessed data. (**c**) Main biological activities of the essential oils from Lauraceae plants. MA: materials (woods, fibres, cork, cane, tannins, latex, resins, gums, waxes, oils, lipids, etc. and their derived products); ME: medicines (both human and veterinary); HF: human food (food, including beverages, for humans only); AF: animal food (forage and fodder for vertebrate animals only); FU: fuels (wood, charcoal, petroleum substitutes, fuel alcohols, etc.—these have been separated from MATERIALS because of their importance); PO: poisons (plants that are poisonous to vertebrates and invertebrates, both accidentally and usefully, e.g., for hunting and fishing); EU: environmental uses (examples include intercrops and nurse crops, ornamentals, barrier hedges, shade plants, windbreaks, soil improvers, plants for revegetation and erosion control, wastewater purifiers, indicators of the presence of metals, pollution, or underground water); GS: gene sources (wild relatives of major crops which may possess traits associated to biotic or abiotic resistance and may be valuable for breeding programs); IF: inverterbrate food (only plants eaten by invertebrates useful to humans, such as silkworms, lac insects and edible grubs, are covered here); SU: social uses (plants used for social purposes, which are not definable as food or medicines, for instance, masticatories, smoking materials, narcotics, hallucinogens and psychoactive drugs, contraceptives and abortifacients, and plants with ritual or religious significance).

As an important economic and ecological tree species, extensive research has been conducted in the fields of phylogeny, isolation, identification, and application of chemical components in the Lauraceae family, investigation and cultivation of Lauraceae plant resources, and endophytic fungi [8,9,10]. According to the *World Checklist of Useful Plant Species* [11] in 2020, Lauraceae comprises the largest number of species utilized for materials and medicinal products, with 361 species identified for material uses and 230 for medicinal purposes (Figure 1a). Notably, the genera *Litsea*, *Cinnamomum*, and *Cryptocarya* exhibit significant clustering in material and medicinal applications (Figure 1b). For instance, *Cinnamomum cassia* Presl, *Cinnamomum burmannii* (Nees et T.Nees) Blume, and *Litsea pungens* Hemsl. are well-known medicinal herbs with a long history of use in traditional Chinese medicine. *Cinnamomum cassia*, *Lindera glauca* (Sieb. et Zucc.) Bl, and *Laurus nobilis* L. are traditional spices that not only enhance the quality and shelf life of meat products but also act as natural antimicrobial agents and antioxidants [12]. The wood of *Cinnamomum camphora* and *Machilus nanmu* (Oliv.) Hemsl. is valued for its hardness and fine grain, making it a preferred material for high-quality construction in shipbuilding, furniture manufacturing, and other industries.

Essential oils play an important role in the material (MA), medicinal (ME), and human food (HF) uses of Lauraceae plants, especially in the *Cinnamomum*, *Laurus*, *Litsea*, and *Lindera*. For instance, *Cinnamomum camphora* (L.) Presl and *Cinnamomum camphora* chvar. Borneol are important essential raw materials for the extraction of camphor and camphor oil and have widespread applications in the chemical and pharmaceutical industries. The essential oils extracted from the branches, leaves, and fruits of *Litsea cubeba* show pharmacological effects, including antibacterial properties and analgesic effects for the treatment of rheumatoid arthritis, as well as antitussive and anticancer activities [13]. Within the genus *Laurus*, the essential oil of *Laurus nobilis* demonstrates exceptional antioxidant capacity alongside biological functions such as antibacterial activity and pest control, positioning it as a viable food preservative [14,15]. The fruit essential oil of *Lindera glauca*—commonly used as a flavoring agent—exhibits potent inhibitory effects against diverse foodborne pathogens, highlighting its potential as a natural antimicrobial agent [16]. The main biological activities and applications of Lauraceae essential oils are shown in Figure 1c.

## 3. Cellular Structures of Lauraceae Producing or Secreting Essential Oils

The synthesis and secretion of essential oils in Lauraceae plants are highly specialized processes involving multi-tissue coordination, complex metabolic pathways, and dynamic organelle changes. The distribution and developmental characteristics of oil cells not only hold taxonomic significance but also serve as a critical gateway for natural product development and investigations into plant adaptability.

**Figure 2:**
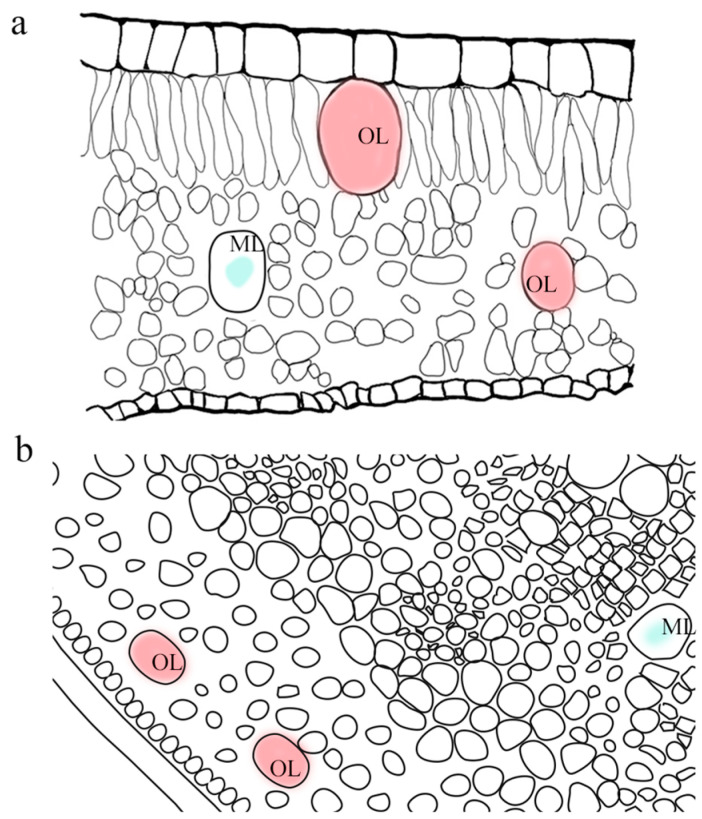
The distribution pattern of the secretory cells. (**a**) A full-expanded leaf. (**b**) A young stem. OL: Oil cell. ML: mucilage cell.

The secretory cells of essential oils in Lauraceae plants include oil cells and mucilage cells [17,18]. Oil cells, typically scattered in distribution, are the main sources of aromatic oils and lipids, while mucilage cells are primarily responsible for the production and storage of mucilage [19]. In Lauraceae plants, secretory cells of essential oils are widely distributed in leaves, stems, roots, flowers, and fruits. According to paraffin sectioning and transparency studies, the distribution of oil cells in the mesophyll of Lauraceae can be classified into four types: Type I, containing only oil cells (e.g., *Cinnamomum*, *Phoebe*); Type II, containing both oil cells and mucilage cells (e.g., *Beilschmiedia*); Type III, containing only mucilage cells; and Type IV, lacking both. These types support the subfamily classification of Lauraceae (Lauroideae mostly exhibit Type I or II, while Cassythoideae tend toward Type III or IV) and reflect their evolutionary trends [20]. Figure 2 shows the locations of secretory cells in the leaf and young stem. When mature, both oil cells and mucilage cells are larger in volume than surrounding cells, and both may exhibit a “cup-shaped structure” in sections. However, oil cells are opaque in paraffin sections and only stain with safranin in safranin-fast green staining; mucilage cells are generally 1-2 times larger in volume than oil cells, mostly appear as a cavity with thin cell walls, and their intracellular components stain deeply with fast green [19].

Oil cell development occurs in four stages: in stage 1, the primary cellulose wall forms, and lipid precursors appear in plastids; in stage 2, a suberized wall layer is deposited on the inner side of the primary wall, enhancing structural stability; in stage 3, the inner cellulose wall layer forms, and vacuoles transform into oil-storage sacs; in stage 4, the cytoplasm degrades, and the oil sac connects to the cell wall via a cup-shaped structure, eventually maturing into an essential oil storage cavity [19,21]. The terpenoid components of essential oils are synthesized through the mevalonic acid (MVA) pathway and the methylerythritol phosphate (MEP) pathway. The products of these pathways are exchanged between organelles via transporter proteins, collectively regulating the diversity of essential oil components. Plastids participate in the early reactions of the MEP pathway, synthesizing terpenoid precursors. The endoplasmic reticulum and Golgi apparatus are involved in lipid transport and the secretion of cell wall components. Vacuoles serve as the primary storage sites for essential oils, releasing their contents through membrane fusion [19].

## 4. The Diversity of Essential Oils in Lauraceae

Based on a review of 75 articles, the essential oil compositions of 18 genera within the Lauraceae family—including *Litsea*, *Cinnamomum*, *Ocotea*, *Aniba*, *Laurus, Lindera*, *Cryptocarya*, *Neocinnamomum*, *Phoebe*, *Machilus*, *Neolitsea*, *Beilschmiedia*, *Cassytha*, *Sassafras*, *Dehaasia*, *Caryodaphnopsis*, *Persea* and *Alseodaphne*—have been systematically summarized (Table S1). Notably, genera such as *Litsea*, *Cinnamomum*, *Laurus, Lindera*, *Cryptocarya*, *Ocotea, Aniba, Neocinnamomum*, *Phoebe* and *Machilus* exhibited relatively higher essential oil extraction rates, while other genera (*Neolitsea*, *Beilschmiedia*, *Cassytha*, *Sassafras*, *Dehaasia*, *Caryodaphnopsis*, *Persea* and *Alseodaphne*) showed lower rates, with the highest values among these latter genera remaining below 1%. Furthermore, significant differentiation in the composition and content of essential oils, particularly concerning the principal components, has been observed across different genera within the Lauraceae family.

### 4.1. Monoterpenoids and Sesquiterpenoids in Lauraceae Essential Oils

The Lauraceae family exhibits significant chemotaxonomic variation in essential oil composition across genera, with distinct patterns of monoterpenoid and sesquiterpenoid accumulation. Some genera within the Lauraceae family may produce essential oils with extremely high proportions of monoterpenoid or sesquiterpenoid. As shown in Table S1, monoterpenoids dominate in genera such as *Litsea*, *Cinnamomum* (leaves/branches), *Laurus*, *Neolitsea*, *Neocinnamomum*, *Sassafras*, and some *Aniba*; whereas sesquiterpenoids are more prominent in *Lindera* (leaves), *Beilschmiedia*, *Cassytha*, *Cryptocarya* (leaves), *Ocotea*, most species of *Machilus*, some *Persea*, and *Alseodaphne*. The specific characteristics of each genus of essential oils are as follows:

There are distinct patterns among the various tissues of the *Litsea* species: fruits contain significant amounts of monoterpenoids, like neral and geranial; leaves of most species are rich in the monoterpenoids, like linalool, 1,8-cineole, and limonene, but *Litsea helferi* and *Litsea viridis* are dominated by sesquiterpenoids, like bicyclogermacrene, β-caryophyllene, and bicycloelemene; flowers and alabastrum are dominated by the monoterpenoids like β-phellandrene and 1,8-cineole [22,23,24,25,26,27,28,29]. In *Cinnamomum*, essential oils from branches and leaves are characterized by high concentrations of monoterpenoids (e.g., camphor, linalool, 1,8-cineole) [30,31,32,33]. In *Neolitsea*, the leaves of *N. sericea* var. *aurata* and *Neolitsea ellipsoidea* primarily contain the monoterpenoid (*E*)-β-ocimene, while *Neolitsea coccinea* is esquiterpenoid-dominated (e.g., selin-11-en-4-α-ol, bicyclogermacrene); fruits mainly consist of monoterpenoids ((*E*)-β-Ocimen, 1,8-cineole) [29,34,35]. In *Lindera*, leaves predominantly contain monoterpenoids (e.g., linalool, camphor) or sesquiterpenoids (e.g., germacrene B, nerolidol, zerumbone); stems and bark are rich in sesquiterpenoids (e.g., cadinol); flowers accumulate sesquiterpenoids (e.g., viridiflorene); and fruits are abundant in monoterpenoids (e.g., (*E*)-β-ocimene and β-ocimene) [16,36,37,38,39,40,41,42,43]. In *Beilschmiedia*, branches and leaves contain rich sesquiterpenoids, including bicyclogermacrene, β-caryophyllene, 9-epi-(*E*)-caryophyllene, δ-cadinene, and germacrene D [29,44,45,46]. Aerial parts of *Cassytha* are enriched in sesquiterpenoids such as spathulenol, β-caryophyllene, (*E*)-nerolidol, bicyclogermacrene, α-humulene, caryophyllene oxide, and germacrene D [47,48]. In *Cryptocarya*, the leaf essential oils are rich in monoterpenoids (e.g., limonene, 1,8-cineole, α/β-pinene, and linalool), or sesquiterpenoids (e.g., bicyclogermacrene, β-caryophyllene, germacrene D) [29,49,50,51,52,53,54,55,56]. Most *Machilus* species’ leaves contain abundant sesquiterpenoids(bicyclogermacrene, β-eudesmol, β-caryophyllene, (*E*)-nerolidol, and α-cadinol), whereas *Machilus japonica* is rich in Monoterpenoids (α-phellandrene, α-pinene, and thymol) [29,57,58,59,60,61]. In *Persea*, leaves are sesquiterpenoid-rich (caryophyllene, (*E*) avocadenynofuran and (*E*)-avocadodienofuran, with fruits and flowers containing sesquiterpenoids like (*E*)-nerolidol [62,63,64,65]. In *Alseodaphne*, the leaves are rich in sesquiterpenoids, ((*E*)-caryophyllene, β-patchoulene, and bicyclogermacren), while fruit are rich in monoterpenoids ((*E*)-β-ocimene) and sesquiterpenoids (*epi*-α-cadinol and δ-cadinene) [55,66,67]. In *Phoebe*, leaves are abundant in monoterpenoids (e.g., α/β-pinene, 1,8-cineole), and its wood contains rich sesquiterpenoids (e.g., agaruspirol, δ-cadinene and (+)-δ-cadinene) [29,68,69]. In *Ocotea*, the leaves are rich in sesquiterpenoids (e.g., germacrene D, β-caryophyllene) [70,71]. In *Aniba*, the leaves, twigs, and branches contain abundant monoterpenoids (e.g., linalool) [63,72,73,74].

Essential oil research within the genera *Laurus*, Sassafras, Dehaasia, and *Caryodaphnopsis* remains limited. Here, we characterize the essential oil composition of one representative species from each genus: *Laurus nobilis* leaves are rich in monoterpenoids (e.g., 1,8-cineole, α-terpinyl acetate, and linalool) [75,76,77], as are those of *Sassafras albidum* (e.g., geranial, neral, and limonene) [78], *Dehaasia cuneata* (e.g., α-pinene, camphene, β-pinene) and *Caryodaphnopsis tonkinensis* (e.g., α/β-pinene) [79]. In *Neocinnamomum*, the bark and leaves primarily contain monoterpenoids such as α/β-pinene and α-phellandrene [80,81].

### 4.2. Other Compounds in Lauraceae Essential Oils

Although terpenoids represent the primary constituents of essential oils, non-terpenoid components play significant roles in aroma characteristics, bioactivity, and functional applications. Non-terpenoid compounds—defined as those not derived from isoprene units (C5)—predominantly include aromatic compounds (terpenoid derivatives and phenylpropanoid derivatives), aliphatic compounds (aldehydes, alcohols, acids, esters), and nitrogen- or sulfur-containing compounds (e.g., sulfides, nitrogenous compounds). In plant essential oils, aromatic compounds represent the second most abundant class of constituents after terpenoids. Lauraceae species such as the *Cinnamomum*, *Persea*, *Machilus*, *Aniba*, and *Cryptocarya* species are rich in phenylpropanoids and esters/lactones (relative content is over 25%). Phenylpropanoids are particularly prominent in *Cinnamomum*, dominating the chemical composition of various tissues, especially bark. For example, *Cinnamomum* roots are rich in phenylpropanoids (e.g., safrole) [30,31,78], as are the bark (e.g., *trans*-cinnamaldehyde, safrole, methyl(*E*)-cinnamate, cinnamaldehyde), branches (e.g., methyl (*E*)-cinnamate), and leaves (e.g., eugenol). Similar compounds occur in *Persea* leaves (e.g., estragole, methyl eugenol) and *Ocotea* leaves (e.g., safrol) [62,63,65,82,83,84,85]. Additionally, leaves of certain species (e.g., *Cinnamomum microphyllum*, *Cinnamomum rhyncophyllum*, *Cinnamomum pubescens*) are dominated by esters (benzyl benzoate) [82]. The genus *Cryptocarya* (e.g., *Cryptocarya massoy* (Oken) Kosterm) contains abundant esters/lactones, such as C-10 and C-12 massoia lactones in the bark and wood [53]. *Persea* leaves are rich in long-chain aliphatic furan derivatives (e.g., 2-(8Z,11Z)-8,11-heptadecadienyl-furan) [63], while *Aniba* leaves, twigs, and branches contain high levels of 1-nitro-2-phenylethane [86].

### 4.3. The Evolutionary History of Essential Oil Composition and Content in Lauraceae Plants

The origin of Lauraceae can be traced back to the Middle and Late Cretaceous [87,88,89]. According to phenology, inflorescence type, anther, fruit, petal, flower character, anther cell number, anther tube development, as well as wood and bark characteristics, different family and genus classification systems have been established within Lauraceae [90]. Early studies on the molecular systematics of Lauraceae, including those by Rohwer [91], Chanderbali et al. [6], and Rohwer & Rudolph [92], produced phylogenetic trees that all featured a terminal branch. The *Persea* group and the *Laureae*-*Cinnamomeae* group, included in this terminal branch, were collectively referred to as core Lauraceae. Therefore, the Lauraceae family is broadly divided into the Core Lauraceae and basal groups. The Core Lauraceae refers to the evolutionarily more advanced lineages within the family, typically forming the main body of Lauraceae comprising multiple highly differentiated tribes or generic groups. These groups exhibit more specialized morphological and molecular characteristics, showing a higher degree of differentiation compared to the basal groups (such as *Cassytha*, *Beilschmiedia*, etc.). The basal Lauraceae refers to the early-diverging lineages within the family that retain more ancestral characteristics, typically positioned at the base of the phylogenetic tree. Although the definition of core Lauraceae is relatively clear, the internal systematic relationships of core Lauraceae remain contentious across different studies. Song et al. [93], through the sequencing and analysis of plastid genomes, highlight a monophyletic Lauraceae, with nine well-supported clades, and further resolve the backbone of the core Laureae in the sense of Chanderbali et al. [6]. In this phylogenomic analysis, the core group included the *Laurus-Neolitsea* clade (*Actinodaphne*, *Iteadaphne*, *Laurus*, *Lindera*, *Litsea*, *Neolitsea*, and *Parasassafras*), *Cinnamomum*-*Ocotea* clade (*Cinnamomum*, *Nectandra*, and *Sassafras*), and *Machilus*-*Persea* clade (*Alseodaphnopsis*, *Dehaasia*, *Machilus*, *Nothaphoebe*, *Persea*, and *Phoebe*), differentiated later than the groups that included the *Beilschmiedia*-*Cryptocarya* clade (*Beilschmiedia*, *Cryptocarya*, *Endiandra*, *Eusideroxylon* and *Syndiclis*), *Cassytha* clade, *Neocinnamomum* clade, and *Caryodaphnopsis* clade.

**Figure 3:**
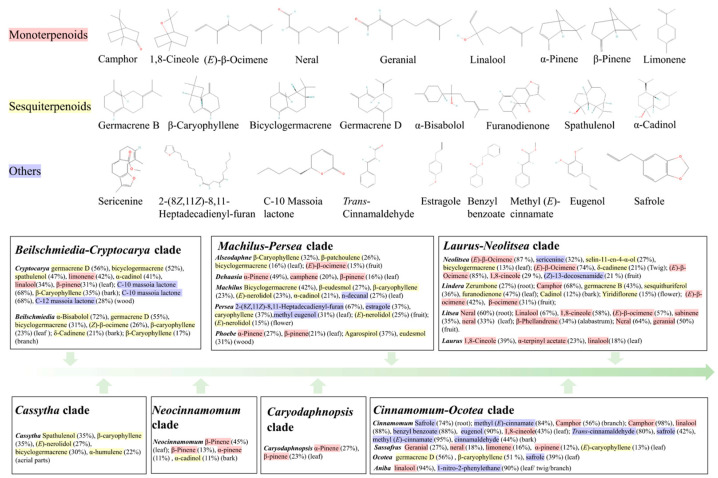
The main chemical components and structures of each genus in the Lauraceae family. The volatile components of each genus in Lauraceae are summarized from the references in Table S1, and the relative content in the figure represents the highest content of the substance that has been identified. The green arrows from left to right indicate the sequence of differentiation time in Lauraceae plants, from early to late. The pink highlights indicate monoterpenoids, the yellow highlights indicate sesquiterpenoids, and the purple highlights indicate other types of compounds.

The plants of the Lauraceae family are widely distributed, and their chemical compositions differ greatly among species belonging to different tribes and genera, especially in terpenoids. Notably, in late-diverging core Lauraceae (e.g., *Cinnamomum*, *Litsea*), essential oils are dominated by specific compounds (e.g., camphor, linalool, 1,8-cineole) in some species (such as *Litsea cubeba* and *Cinnamomum camphora*), revealing distinct chemotypes within the group. However, the *Cassytha*, one of the basal lineages of Lauraceae, has not been found to contain a single dominant compound in its essential oils, which typically consist of a mixture of various terpenes (Figure 3). It has been suggested that when the production of new metabolites provides an adaptive advantage to the plant, such production is maintained and/or increased [1]. In radiating species and those with slower evolutionary rates, specialized terpenoids often exist as mixtures of multiple related compounds, indicating that terpenoid diversity may confer ecological advantages [94]. As the primary components of plant essential oils, terpenoids fulfill crucial defensive and non-defensive roles, and coevolution with natural enemies may drive the continuous emergence of new terpenoids within specific plant lineages [94]. Rapid diversification in core Lauraceae (likely linked to ecological expansion, e.g., tropical to subtropical niches) led to divergent selection pressures (e.g., herbivores, pollinators, climate) [95]. This drove chemical specialization, where different populations evolved distinct dominant compounds. For example, *Cinnamomum camphora* populations may produce either camphor (strong insect repellent) or linalool (pollinator attraction), promoting speciation via niche partitioning. In addition, gene duplication or functional divergence in terpene synthases (e.g., camphor synthase) enabled the efficient production of specific compounds, providing ecological advantages that fueled further diversification. For example, if a compound (e.g., safrole) becomes too common, herbivores may develop resistance, favoring alternative chemotypes (e.g., 1,8-cineole) and maintaining chemical polymorphism. As an early-diverging lineage, *Cryptocarya* may retain an ancestral “generalist” strategy—producing diverse terpenes to deter a broad range of herbivores/pathogens, rather than specializing; and the limited genetic variation in key enzymes (e.g., cytochrome P450s) could restrict chemical innovation, resulting in less pronounced chemotype differentiation.

## 5. Genetic and Biochemical Bases of Essential Oil Terpenoids in Lauraceae

Terpenoids, recognized as one of the most abundant and widely distributed classes of natural products, are synthesized by all forms of life. Hundreds of terpenoids are present in nearly all plant species, where they are categorized as primary metabolites. However, a significant proportion of terpenoids are restricted to specific lineages or even species, serving as adaptations to ecological environments; these are termed specialized terpenoid metabolites. Genes involved in plant secondary metabolism often exhibit a random distribution throughout the plant genome, complicating the identification of all genes associated with biochemical pathways underlying plant-specific metabolism. The increasing availability of plant genomes at the chromosomal level provides new opportunities to investigate the evolutionary origins and metabolic processes of plant-specific terpene synthesis. Genomics and metabolomics have established a bridge between terpenoid essential oil components and their corresponding genes. The production of terpenoids has been extensively documented across diverse plant lineages, with a notable association identified in the Lauraceae lineage.

### 5.1. Terpenoids Biosynthesis Pathway

**Figure 4:**
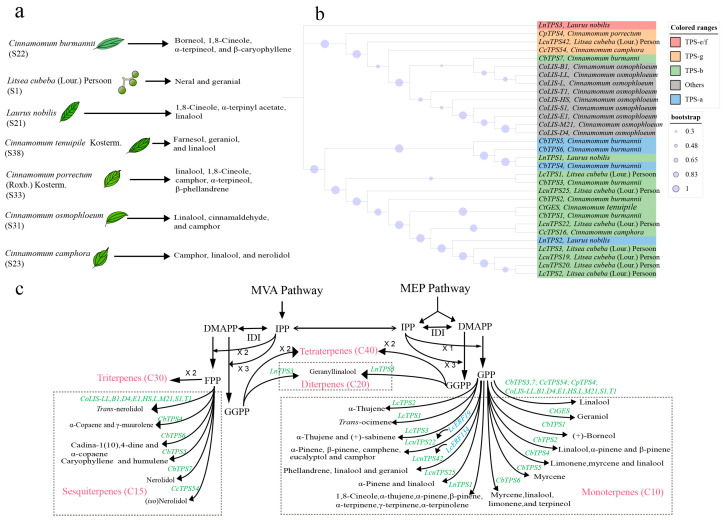
Key genes for essential oil synthesis in Lauraceae. (**a**) Main components of essential oil in Lauraceae species where key genes are located. S1, S21, S22, etc., are the corresponding numbers of the species in Table S1. (**b**) Phylogenetic tree of the *TPS* genes. Based on MEGA 11.0.13 software, the MUSCLE method was used to perform the alignment of TPS amino acid sequences (Table S2). The tree was constructed using the neighbor-joining method with the bootstrap value set to 1000. (**c**) Plant terpene biosynthesis pathway. The dashed box contains the terpene biosynthetic pathway of Lauraceae. *TPS* genes are in green and transcription factors are in blue.

The biosynthesis of plant terpenoids is generally divided into four stages: the synthesis of the terpenoid universal precursor isopentenyl pyrophosphate (IPP) and its double bond isomer dimethylallyl pyrophosphate (DMAPP) through the MVA pathway and the MEP pathway; the generation of direct precursor substances geranyl diphosphate (GPP), farnesyl pyrophosphate (FPP), and geranylgeranyl pyrophosphate (GGPP); the synthesis of monoterpenes (C10), sesquiterpenes (C15), and diterpenes (C20) skeletons from GPP, FPP, and GGPP catalyzed by terpene synthase [2,96] (Figure 4c); and the diversification of terpenoid backbones through oxidation, methylation, acylation, or glycosylation via tailoring enzymes—such as cytochrome P450 oxygenases (CYPs), dehydrogenases, methyltransferases, acyltransferases, and glycosyltransferases. As the terminal and most pivotal enzymes governing terpenoid backbone formation, terpene synthases (TPSs) have attracted extensive research attention due to their central roles in terpenoid biosynthesis.

### 5.2. Terpene Synthase (TPS) Gene Family Size in Lauraceae Occurs Significantly Expansion

Plant genome sequencing has been carried out for many years, and the genome sequencing of Lauraceae species such as *Cinnamomum camphora* [97,98,99,100,101], *Cinnamomum chago* [102], *Cinnamomum kanehirae* [103], *Litsea cubeba* [104], *Lindera megaphylla* [105], *Lindera glauca* [106], *Phoebe bournei* [107], and *Persea americana* [108,109] has been conducted in recent years. To date, whole-genome analyses across diverse plant lineages have revealed that typical *TPS* genes are encoded by medium to large gene families. Except for *Physcomitrella patens*—whose genome contains only one *TPS* gene—most plant TPS gene families include 20 to 170 members. For example, *Eucalyptus*, *Vitis vinifera*, *Populus tomentosa*, *Oryza sativa*, and *Arabidopsis thaliana* have 113, 69, 38, 32, and 32 *TPS* genes, respectively.

**Figure 5:**
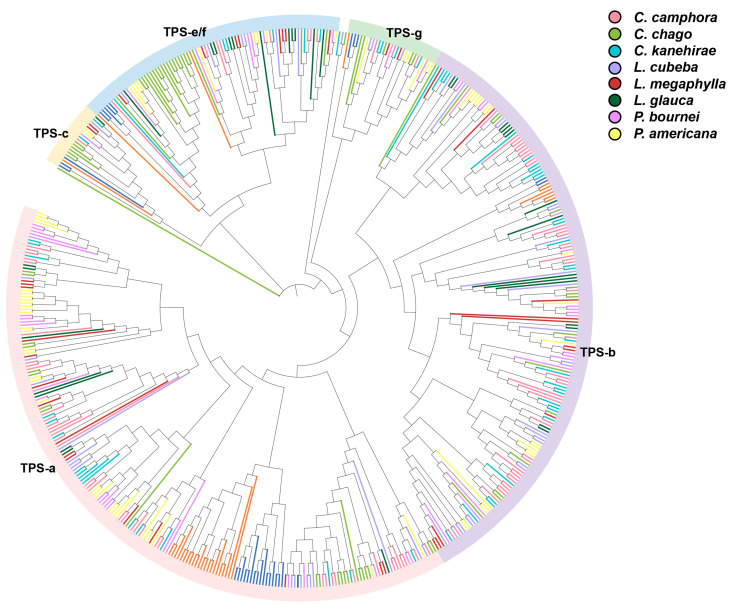
Phylogeny of *TPS* genes. Phylogenetic analysis was carried out using the maximum likelihood method. Bootstrap values are shown as a percentage of 1000 replicates. Phylogenetic analysis was conducted on the TPS gene families of 8 Lauraceae species and 2 other plant species.

We have analyzed the TPS gene families of eight Lauraceae species with well-sequenced and annotated genomes: *Cinnamomum camphora*, *Cinnamomum chago*, *Cinnamomum kanehirae*, *Litsea cubeba*, *Lindera megaphylla*, *Lindera glauca*, *Phoebe bournei*, and *Persea americana* (Table 1). The number of *TPS* genes in these Lauraceae species exhibits considerable variation, ranging from 35 to 93 members. Notably, *Cinnamomum camphora* possesses the largest TPS gene family (93 members) among the analyzed species, which likely underlies its remarkable capacity for producing diverse terpenoid compounds. For comparison, we also included the *Arabidopsis thaliana* and *Zea mays* genome in our analysis. Phylogenetic analysis showed that the TPS gene family of Lauraceae species could be divided into five subfamilies, and the Lauraceae TPS gene family had formed independent clades in the TPS-a, TPS-b, TPS-g, TPS-e/f, and TPS-c subfamilies (Figure 5). Members of the TPS-a subfamily are responsible for the synthesis of sesquiterpenes. *Phoebe bournei* (S66) and *Persea americana* (S59) possessed the highest number of *TPS-a* genes within their respective TPS gene families, aligning with the dominance of sesquiterpenoids in their essential oil compositions. The TPS-b subfamily was the most abundant in *Cinnamomum camphora* (S23), *Cinnamomum kanehirae* (S28), and *Litsea cubeba* (S1), potentially contributing to the rich monoterpene synthesis in *Cinnamomeae* and *Litsea*.

### 5.3. Gene Duplication Offers Dynamic for Diversity of Terpenoids in Lauraceae

Mechanisms underlying the formation and evolution of plant volatiles include (1) gene duplication events that preserve original enzymatic functions while allowing duplicated genes to evolve new functions; (2) convergent evolution, where new functions arise independently across multiple lineages; (3) evolution of non-duplicated existing genes to acquire new enzymatic functions while losing original functions; and (4) loss of enzymatic activity [1]. During plant volatile formation, enzymatic functional diversity can emerge with minimal changes in enzyme structure and may be further enhanced by exposure to variable environments (e.g., constitutive or inducible conditions). TPSs diversity directly influences terpenoid diversity: a single TPS can catalyze different substrates to generate distinct terpenoids, while different TPSs may catalyze the same substrate to produce varied terpenoids.

In the evolutionary process of Lauraceae, genome-wide duplication, tandem duplication events, and segmental duplication events may promote the expansion of the TPS gene family, thereby driving the diversification of TPSss and the production and diversification of terpenoids. Genome rearrangement events in *Cinnamomum kanehirae* may have contributed to the diversification of TPSss and the subsequent clustering of genes involved in terpenoid biosynthesis [103]. In *Litsea cubeba* [104], tandem repeat events drove the expansion of the TPS-b subfamily, leading to terpenoid accumulation. In *Cinnamomum camphora* [97,99,100,101], both tandem and segmental duplication events significantly promoted the expansion of the TPS gene family. And the TPS-a and TPS-b subfamilies exhibited the highest diversity, potentially facilitating the biosynthesis of monoterpenoids and sesquiterpenoids [99]. In *Phoebe bournei* [107], lineage-specific duplication and contraction events were prevalent across TPS subfamilies, leading to significant variation in terpenoid profiles among Lauraceae species. Lineage-specific terpenoids, which have evolved throughout the history of green plants, are generally hypothesized to function in plants’ ecological interactions with both biotic and abiotic components of their environment—such as a defense against herbivores and pathogens, as well as signals and rewards for beneficial organisms [94]. Therefore, the new functions obtained by the expansion and differentiation of the TPS gene family may be related to the specific terpenoids formed by plants to adapt to the environment.

Differences in gene expression or protein function promote the retention of duplicated genes. Studies have demonstrated that mechanisms such as positive selection, changes in protein subcellular localization, and variations in promoter regions regulate the differential expression, retention, and functional diversification of duplicated genes [110,111]. Additionally, recent studies have demonstrated that genes involved in terpene biosynthesis often form gene clusters [112,113]. These plant metabolic gene clusters are defined as assemblies of two or more non-homologous genes that are closely linked and encode enzymes participating in biosynthesis, transporters, and cofactor synthases [113]. Therefore, expansion of the terpenoid synthase gene family, formation of gene clusters, differentiation of gene functions, and variations in gene regulation may collectively drive changes in terpenoid production and composition in Lauraceae [104].

### 5.4. Function and Regulatory Mechanism of TPSs in Lauraceae

Every plant species harbors numerous genes involved in terpene biosynthesis within its genome, forming the genetic basis for the capacity of modern plant lineages to produce abundant terpenes [94]. Significant progress has been made in elucidating the terpenoid biosynthesis pathways in Lauraceae. Consequently, the production and modification stages of terpenoids—which govern terpenoid diversity—represent critical frontiers in current research on plant terpenoid metabolism [114].

*TPS* genes involved in terpenoid biosynthesis have been cloned from terpene-rich plants. Based on seven publications (2004–2022), we summarized the *TPS* genes in the Lauraceae family. *TPS* genes from *Cinnamomum*, *Litsea*, and *Laurus* have been reported (Figure 4a), with functional analyses conducted in both *Escherichia coli* and allogeneic plant hosts (Table S2). The *TPS* genes related to terpenoid biosynthesis encompass the TPS-a, b, g, and TPS-e/f subfamilies, with the majority of reported genes belonging to the TPS-b subfamily. These genes catalyze the conversion of GPP or FPP substrates to produce single or multiple terpenes, respectively. Phylogenetic analyses (Figure 4b,c) revealed that *TPS* genes with distinct functions—such as those regulating the synthesis of linalool, α-thujene, nerolidol, and α-pinene—clustered into separate clades. This pattern indicates that *TPS* genes with similar functions exhibit high sequence homology. Additionally, while single-gene overexpression can modestly enhance target terpenoid synthesis, achieving substantial increases in final yield remains challenging [115].

Transcription factors can simultaneously regulate multiple synthetase genes within a metabolic pathway, offering significant potential to enhance the yield of targeted specialized terpenes. This approach serves as an effective strategy to substantially increase end products at the molecular level [116]. Within the Lauraceae family, researchers have utilized the transcription factors *LcERF19* and *LcERF134* to induce the expression of *LcTPS42* in *Litsea cubeba*, thereby improving the synthesis of essential oil terpenoids [117,118].

## 6. Discussion

Influenced by different geographical environmental factors and extraction techniques, significant differences may exist in the chemical composition and quality of plant essential oils extracted from the same parts of the same plant. Under the multidimensional effects of climatic factors, the synthesis of essential oils changes. For example: low temperatures can increase the proportion of camphor in the leaves of *Cinnamomum camphora* [97], while high light intensity significantly increases the proportion of 1,8-cineole in the essential oil of *Cinnamomum longepaniculatum* [119]. Altitude differentiation leads to differences in component types. For instance, low altitudes (<500 m) promote the synthesis of oxygenated monoterpenes, while high altitudes (>1000 m) increase sesquiterpenes and oxidation derivatives [120]. The complex regulation of soil moisture and nutrients—such as changes in elements like nitrogen, phosphorus, and magnesium—can cause variations in components or yields. Extraction techniques have selectivity and limitations on the yield and composition of essential oils [121]. The traditional steam distillation extraction method can obtain rich essential oil components, but it may cause potential loss or destruction of volatile compounds, and the essential oil yield is low [122]. Supercritical fluid CO_2_ extraction can obtain abundant terpenes, but the yield of polar oxygen-containing compounds is low, and the types of essential oils obtained are fewer [4]. The organic solvent method for extracting essential oils can achieve high yields of specific products, but it has the problems of low oil yield and organic solvent residues [123]. Due to the wide diversity of Lauraceae plant species, coupled with the varying distributions of their oil cells and distinct extraction objectives, the selection of extraction methods should be guided by functional orientation.

Throughout the lengthy process of evolution, the essential oils of the Lauraceae family have diversified as a result of interactions between genetic and environmental factors. Geological history and lineage differentiation, ecological adaptation and natural selection, environmental stress responses, and evolutionary mechanisms at the genomic level are the evolutionary factors driving the diversity of essential oils [124]. Whole genome sequencing serves as a vital foundation for the functional analysis, expression regulation, and genetic mapping of genes associated with important plant traits. Notably, the information regarding the sequence, structure, and evolution of structural and regulatory genes involved in essential oil synthesis is crucial for understanding the genetic background of terpene-rich plants, particularly aromatic species [101]. Based on the joint analysis of multi-omics data such as genomics, transcriptomics, and metabolomics, researchers have identified key genes involved in the synthesis of important terpenoid compounds in Lauraceae and mapped the anabolic pathways [124]. The gene clusters for plant secondary metabolite synthesis are one of the iconic features of the genome. They can operate as efficiently as operons in prokaryotic genomes. The arrangement of genes involved in the same metabolic pathway in a clustered manner can greatly promote rapid stress responses in plants and is conducive to the co-regulation, synergistic action, and co-inheritance of genes. The gene cluster related to citral biosynthesis (MYB44-ADH28/ADH29 gene cluster) has been identified in *Litsea cubeba* [125]. This gene cluster forms a species-specific metabolic pathway through the duplication-divergence mechanism, while the transcriptional regulatory network (such as the *MYB44* repression model) achieves precise environmental responses.

Currently, conventional and molecular breeding approaches targeting the essential oil-rich traits of Lauraceae have been applied; however, stable production resources for most high-oil-content Lauraceae species remain limited, with exceptions with commercially cultivated genera such as *Litsea*, *Cinnamomum*, *Persea*, and *Laurus*. Quality-focused genetic improvement represents a critical frontier in modern tree breeding. Using molecular biology and genetic engineering techniques to precisely enhance terpene yield is a promising direction for the genetic improvement of essential oil species. Key steps for enhancing essential oil production in Lauraceae plants via genetic engineering include (1) targeted gene screening: identification and isolation of key genes (e.g., *TPS*, *DXS*) through transcriptome sequencing or homology-based cloning; (2) vector construction: development of high-efficiency expression vectors (e.g., CRISPR/Cas9 or overexpression systems); (3) transient transformation: rapid functional validation using Agrobacterium-mediated transient expression systems, combined with molecular detection (PCR/qPCR) and phenotypic analysis (GC-MS for terpenoid profiling); and (4) stable transformation and breeding: stable genetic modification via tissue culture, followed by multi-generational screening to develop elite varieties with optimized essential oil traits.

## 7. Prospect

Lauraceae plants are highly diverse, leading to significant variability in their essential oils. Therefore, it is essential to explore more species with potential value for essential oil utilization, including component identification, extraction methods, and biological activity analysis. Additionally, the evaluation system for Lauraceae plant essential oils should be further improved by establishing standard fingerprint profiles of essential oil components for more species, providing a quality assessment basis for their applications. Furthermore, the mechanisms of action of essential oils should be further investigated to develop related products in fields such as food, flavors and fragrances, and pharmaceuticals.

Among the core branches of Lauraceae, *Cinnamomum*, *Litsea*, and *Laurus*, along with *Cryptocarya* from the basal group, exhibit higher extraction rates of essential oil. Notably, the dominant component in the essential oils of *Cinnamomum*, *Litsea*, and *Laurus* can exceed 90%. While *Cryptocarya* also shows high essential oil yields, it lacks a single prominent major component. Therefore, there is a critical need to advance the precision cultivation of Lauraceae species to promote the development and utilization of their essential oil resources. For example, the targeted improvement of essential oil content and specific component ratios (e.g., camphor, citral) in Lauraceae can be achieved through strategies such as varietal breeding, cultivation optimization, and genetic engineering, complemented by analytical techniques including GC/MS, HPLC, and TLC to enable precision-driven exploitation of essential oil resources.

At present, whole genome sequencing has been carried out in some Lauraceae plants, but limited to the species of *Persea* [108], *Cinnamomum* [97,99,100,101,103,126], *Litsea* [104], *Phoebe* [107,127], and *Lindera* [106]. The identification of functional genes in the genomes of Lauraceae plants involves rich content, but the overall research is not deep enough, still remaining at the level of gene identification and metabolic pathway analysis, with few functional validations conducted. Transcription factor families such as WRKY, APETALA2/ethylene responsive factor (AP2/ERF), basic Helix-Loop-Helix (bHLH), and MYB have been implicated in plant terpenoid metabolism, yet they are seldom reported in Lauraceae. Similarly, the research on the gene clusters for secondary metabolite synthesis in Lauraceae plants is still in its infancy. Therefore, future whole-genome analyses should cover a broader range of Lauraceae species and adopt multi-omics approaches including resequencing, transcriptomics, metabolomics, and proteomics to further clarify the genetic information of Lauraceae plants. Meanwhile, it is necessary to validate more functional genes related to the synthesis of essential oil components in Lauraceae and conduct in-depth research on transcription factor regulatory networks and gene cluster analysis.

At present, the functional validation of genes in Lauraceae plants primarily relies on heterologous plant hosts, and stable genetic transformation technologies are lacking. Therefore, overcoming barriers to genetic transformation and establishing a reliable genetic transformation system for Lauraceae plants represent important breakthroughs to be achieved in the future.

## Figures and Tables

**Table 1:** Sizes of the TPS gene family and subfamilies in seven model plant genomes.

Species	Genome Size	*TPS* Numbers	TPS Subfamily					
			a	b	c	e/f	g	x
*Cinnamomum camphora*	706.47 Mb	93	38	43	1	8	3	0
*Cinnamomum chago*	1061.15 Mb	72	22	15	7	22	5	1
*Cinnamomum kanehirae*	730.7 Mb	60	17	30	2	6	4	1
*Litsea cubeba*	1325.69 Mb	52	17	24	1	6	3	1
*Lindera megaphylla*	1268.6 Mb	39	16	11	2	8	2	0
*Lindera glauca*	2092.2 Mb	35	8	15	1	11	0	0
*Phoebe bournei*	1065.88 Mb	53	25	19	1	6	2	0
*Persea americana*	841.6 Mb	69	32	20	2	9	6	0

Note: The identification of the TPS gene family was conducted using the HMM models PF01397 and PF03936 of the TPS gene family as the model alignment sequences (e-value < 10-10), and sequences with sequence lengths greater than 200 aa were screened.

## Data Availability

Data are contained within the article.

## Supplementary Materials

The following supporting information can be downloaded at: https://www.mdpi.com/article/10.3390/ijms26125690/s1. References [128,129,130,131,132,133,134,135,136,137,138,139,140,141,142,143] are cited in the Supplementary Materials.
