# Supplementary material for: An Updated Review on Essential Oils from Lauraceae Plants: Chemical Composition and Genetic Characteristics of Biosynthesis
Source: Int J Mol Sci. 2025 Jun 13;26(12):5690. doi: 10.3390/ijms26125690 (PMC12192576; doi:10.3390/ijms26125690)
Supplement: Supplementary file 1 [file ijms-26-05690-s001.zip › ijms-3669053-supplementary.pdf]

Supplementary Materials

Fanglan Wu [1,2,3,4], Yicun Chen [1,3], Ming Gao [1,3], Wei Li [1,4], Yunxiao Zhao [1,3,*] and Yangdong Wang [1,3,*]

Table S1. Main constituents of Lauraceae essential oil.

Clade	Genera	Specie	Tissue	oil yield (%)	Monoterpenoid (%)	sesquiterpenoid (%)	Other compound (%)	Major constituent	Reference
Laurus-Neolitsea clade	*Litsea*	[S1]*L. cubeba*	Root	<1	84	7	77	Monoterpenoids: neral (22%), citronellal (9%), linalool (7%), isopulegol (6%), β-phellandrene (5%)	[1]
			Alabastrum	2	95	4	-	Monoterpenoids: β-phellandrene (34%), 1,8-cineole (11%), α-pinene (9%), β-pinene (8%)	
			Flower	3	95	3	-	Monoterpenoids: 1,8-cineole (14%), α-pinene (8%), β-pinene (7%)	
			Fruit	2	88	1	9	Monoterpenoids: neral (64%), limonene (7%)	
			Leaf	1	59	27	13	Monoterpenoids: 1,8-cineole (14%), linalool (7%), limonene (7%); Sesquiterpenoids: γ-elemene(8%), caryophyllene (8%)	
			Stem	<1	92.99	5	1	Monoterpenoids: β-phellandrene (19%), terpinene-4-ol (12%), limonene (10%), α-thujano1 (9%), β-pinene (7%)	
			Fruit	5	96	1	1	Monoterpenoids: neral (37%), geranial (44%), citronella (9%)	[2]
			Leaf	2	86	11	-	Monoterpenoids: linalool (67%), limonene (6%), neral (6%); Sesquiterpenoids (6%): β-bisabolene (6%)	
			Fruit	4	99	1	-	Monoterpenoids: neral (31%), geranial (37%), limonene (11%)	[3]
			Leaf	14	98	2	-	Monoterpenoids: 1,8-cineole (58%), sabinene (12%), α-terpinyl acetate (10%)	
			Fruit	3- 5	94–98	0-3	0-1	Monoterpenoids: neral (34-37%), geranial (44-50%), D-limonene (0-5%)	[4]

		Leaf	1	83	4	3	Monoterpenoids: sabinene (14%), limonene (9%), linalool (10%), neral (33%)	[5]
		Stem	-	98	1	1	Monoterpenoids: sabinene (10%), limonene (13%), neral (53%)	
		Fruit	-	97	-	3	Monoterpenoids: limonene (7%), terpinen-4-ol (5%), neral (66%)	
		Root	-	93	1	3	Monoterpenoids: limonene (14%), neral (60%)	
	S2*L. elongata* var. *subverticillate*	Leaf	<1	9	38	52	Monoterpenoids: (1R)-α-pinene (7%); Sesquiterpenoids: α-bergamotene (8%), α-caryophyllene (7%)	[6]
	S3*L. ferruginea*	Leaf	<1	85	12	2	Monoterpenoids: sabinene (35%), α-pinene (10%), γ-terpinene (8%), limonene (7%), terpinen-4-ol (7%), α-terpinene (5%), myrcene (5%)	[5]
	S4*L. glaucescens*	Leaf	1	84	4	7	Monoterpenoids: 1,8-cineole (26%), o-cymene (26%), limonene (9%), tepinen-4-ol (5%)	[7]
	S5*L. glutinosa*	Leaf	1	86	9	1	Monoterpenoids: (*E*)-β-ocimene (57%), α-pinene (7%), β-pinene (8%)	[5]
	S6*L. helferi*	Leaf	<1	48	48	6	Monoterpenoids: limonene (18%), α-phellandrene (8%); Sesquiterpenoids: β-caryophyllene (14%), bicyclogermacrene (13%), bicycloelemene (12%)	
	S7*L. mollis*	Leaf	1	14	30	56	Monoterpenoids: D-limonene (8%); Sesquiterpenoids: *trans*-geraniol (7%), β-elemene (8%)	[7]
	S8*L. verticillata*	Leaf	<1	78	17	-	Monoterpenoids: α-pinene (26%), linalool (23%), β-pinene (12%)	[5]
	S9*L. viridis*	Leaf	<1	24	50	19	Monoterpenoids: α-pinene (11%), β-pinene (8%); Sesquiterpenoids: bicyclogermacrene (26%); Others: decanal (14%)	[8]
Neolitsea	S10*N. buisanensis* Yamam. & Kamik.	Leaf	<1	25	37	25	Fatty acids: hexadecanoic acid (12%); Amides: (*Z*)-13-docosenamide (8%)	[9]
		Stem	<1	44	15	32	Monoterpenoids: limonene (10%); Diterpenoids: phytol (13%); Amide: (*Z*)-13-docosenamide (13%)	

		Fruit	<1	60	7	31	Monoterpenoids: 1,8-cineole (29%), sabinene (15%), *α*-terpineol (9%); Amide: (*Z*)-13-docosenamide (21%)	
	S11*N. coccinea*	Stem	<1	-	85	-	Sesquiterpenoids: δ-cadinene (21%), 1-*epi*-cubenol (11%), cyperotundone (11%)	[10]
		Leaf	<1	1	90	-	Sesquiterpenoids: selin-11-en-4-*α*-ol (27%), bicyclogermacrene (13%), γ-eudesmol (7%), germacrene D (6%), globulol (6%)	
	S12*N. ellipsoidea*	Leaf	<1	94	3	-	Monoterpenoids: (*E*)-β-ocimene (88%)	[8]
	S13*N. sericea* var. aurata	Leaf	1	52	48	-	Monoterpenoids: (*E*)-β-ocimene (49%); Other: sericenine (32%)	[11]
Lindera	S14*L. chunii* Merr.	Flower	-	3	91	2	Sesquiterpenoids: viridiflorene(15), β-cadinene (10%), germacrene B (6%), globulol (6%), τ-cadinol (5%), γ-muurolene (5%)	[12]
		Leaf	-	1	93	2	Sesquiterpenoids: germacrene B (43%), globulol (12%), ledol (11%)	
		Stem	-	2	93	2	Sesquiterpenoids: *α*-cadinol (9%), globulol (8%), τ-cadinol (7%), viridiflorene (6%)	
	S15*L. glauca*	Fruit	2	57	40	2	Monoterpenoids: β-ocimene (31%)	[13]
		Fruit	<1	62	31	3	Monoterpenoids: (*E*)-β-ocimene (42%), 3-carene (6%), 1,8-cineole (4%); Sesquiterpenoid: *α*-copaene (13%), δ-cadinene (6%)	[14]
	S16*L. obtusiloba* Blume	Bark	-	12	66	20	Monoterpenoids: terpinolene (6%); Sesquiterpenoids: β-cadinol (12%), hedycaryol (10%), β-eudesmol (10%), caryophyllene (6%), τ-cadinol (6%), τ-eudesmol (5%)	[15]
	S17*L. pulcherrima*	Leaf	-	-	97	-	Sesquiterpenoids: furanodienone (47%), curzerenone (18%)	[16]
	S18*L. rufa* Hook. f.	leaf	1	97	1	-	Monoterpenoids: camphor (68%), limonene (7%), *α*-pinene (7%)	[17]
	S19*L. setchuenensis*	Leaf	<1	1	7	71	Fatty acids: palmitic acid (27%), linoleic acid (5%); Aldehydes: *cis*-13-Octadecenal (5%), myristic aldehyde (7%); Diterpenoids: phytol (7%)	[18]

		[S20]*L. strychnifolia* (Sieb. and Zucc.) F. Villars	Leaf	<1	16	78	1	Monoterpenoids: 1,8-cineole (5%); Sesquiterpenoids: sesquithuriferol (36%), 14-oxy-*α*-muurolene (17%)	[19]
			Leaf	<1	16	78	1	Monoterpenoids: 1,8-cineole (5%); Sesquiterpenoids: sesquithuriferol (36%), 14-oxy-*α*-muurolene (16%)	[20]
			Root	<1	46	45	-	Monoterpenoids: geranyl acetate (12%), (*E*)-β-ocimene (10%), 1,8-cineole (8%); Sesquiterpenoids: zerumbone (27%)	
	Laurus	[S21]*L. nobilis*	Leaf	1	68	4	15	Monoterpenoids: 1,8-cineole (25%), linalool (18%), camphene (7%); Phenylpropanoid: eugenyl-methylether (12%); Aldehyde: isovaleraldehyde (10%)	[21]
			Leaf	<1	76	6	15	Monoterpenoids: 1,8-cineole (35%), linalool (3%), camphene (9%), β-phellandrene (6%); Aldehyde: isovaleraldehyde (9%),	
			Leaf	1	71	1	18	Monoterpenoids: 1,8-cineole (39%), linalool (9%), *α*-terpineol (6%), 2-carene (6%); Aldehyde: isovaleraldehyde (10%)	
			Leaf	1	83	3	6	Monoterpenoids: 1,8-cineole (32%), sabinene (12%), linalool (10%), *α*-terpinyl acetate (6%), *α*-pinene (6%)	[22]
			Leaf	1	84	3	9	Monoterpenoids: 1,8-cineole (31%), *α*-terpinyl acetate (15%), *α*-terpineol (8%), sabinene (8%), terpinen-4-ol (6%), *α*-pinene (5%)	[23]
			Leaf	5	86	2	10	Monoterpenoids: 1,8-cineole (29%), *α*-terpinyl acetate (23%), sabinene (12%), *α*-pinene (6%); Phenylpropanoids: methyl-eugenol (8%)	
Cinnamomum - *Ocotea* clade	*Cinnamomum*	[S22]*C. burmannii*	Leaf	<1	48	24	6	Monoterpenoids: borneol (43%); Sesquiterpenoids: caryophyllene (10%)	[24]
			Bark	<1	25	58	6	Monoterpenoids: 1,8-cineole (8%), camphor (6%); Sesquiterpenoids: 1-isopropyl-4,7-dimethyl-1,2,3,5,6,8a-hexahydronaphthalene (12%), γ-muurolene (9%), *α*-	

						muurolene (7%), α-terpineol (7%), δ-selinene (6%), α-ylangene (5%)	
	Branch	<1	47	22	6	Monoterpenoids: borneol (44%); Sesquiterpenoids: caryophyllene (6%)	
	Leaf	1	-	-	-	Monoterpenoids: borneol (19-29%), 1,8-cineole (0-24%), α-terpineol (6-10%), α-pinene (0-5%); Sesquiterpenoids: spathulenol (2-10%), β-caryophyllene (2-9%)	[25]
S23*C. camphora* (L.) Presl.	Leaf	2	81	-	1	Monoterpenoids: camphor (69%)	[26]
	New branch	<1	77	3	2	Monoterpenoids: camphor (56%), 1,8-cineole (6%)	
	Old branch	<1	75	3	2	Monoterpenoids: camphor (53%), 1,8-cineole (8%)	
	Stem	<1	67	-	21	Monoterpenoids: camphor (51%), 1,8-cineole (7%); Phenylpropanoids: safrole (21%)	
	Tap root	2	11	-	74	Monoterpenoids: camphor (9%); Phenylpropanoids: safrole (73%)	
	Lateral root	2	10	-	72	Monoterpenoids: camphor (8%); Phenylpropanoids: safrole (74%)	
	Leaf	3	94	-	-	Monoterpenoids: camphor (93%)	[27]
	Branch	2	62	7	-	Monoterpenoids: camphor (54%), limonene (7%), α-pinene (7%)	
	Wood	1	83	2	5	Monoterpenoids: camphor (53%), 1,8-cineole (20%), α-terpineol (6%)	
	Root	2	37	1	59	Monoterpenoids: 1,8-cineole (18%), camphor (12%); Phenylpropanoids: safrole (58%)	
	Leaf	1	96	1	1	Monoterpenoids: 1,8-cineole (41%), linalol (21%), β-phellanderene (10%), α-terpineol (10%)	[28]
S24C. *cordatum*	Leaf	3	55	7	39	Monoterpenoids: β-phellandrene (9%), linalool (17%), terpinen-4-ol (7%); Phenylpropanoids: methyl(*E*)-cinnamate (17%); Esters: benzyl benzoate (8%)	[29]
S25*C. cassia*	Bark	-	5	11	83	Phenylpropanoids: *trans*-cinnamaldehyde (80%)	[30]
	Bark	2	10	2	84	Phenylpropanoids: *trans*-cinnamaldehyde (78%), 2-methoxycinnamaldehyde (9%)	[31]

S26C. i*mpressicostatum*	Leaf	3	20	2	78	Esters: benzyl benzoate (51%), benzyl salicylate (8%)	[29]
	Bark	2	3	-	97	Phenylpropanoids: methyl (*E*)-cinnamate (86%)	
	Twig	1	10	2	89	Monoterpenoids: α-terpinene (8%); Phenylpropanoids: methyl (*E*)-cinnamate (68%); Esters: benzyl benzoate (8%)	
S27*C. japonicum*	Leaf	1	79	5	13	Monoterpenoids: 1,8-cineole (25%), borneol (25%), o-cymene (12%), α-phellandrene (7%)	[28]
S28C. *kanehirae*	Leaf	1	36	4	34	Monoterpenoids: 3-thujene (5%), limonene (7%), linalool (11%); Others: 4-isopropyltoluene (17%)	[32]
	Leaf	1	86	2	1	Monoterpenoids: α-pinene (5%),α-phellandrene (17%), 1,8-cineole (55%), α-terpineol (8%)	
	Leaf	<1	81	4	5	Monoterpenoids: linalool (66%)	
	Leaf	1	10	39	40	Monoterpenoids: linalool (6%); Sesquiterpenoids: β-caryophyllene (14%), β-eudesmene (10%); Aldehydes: tetradecanal (23%)	
S29C. *microphyllum*	Leaf	5	35	3	63	Esters: benzyl benzoate (88%)	[29]
S30C. *mollissimum*	Leaf	1	1	-	99	Esters: benzyl benzoate (88%)	
S31*C. osmophloeum* Kanehira	Leaf	-	-	-	-	Monoterpenoids: linalool (40%), camphor (9%); Phenylpropanoids: cinnamaldehyde (7%); Esters: *(E)*-cinnamyl acetate (12%)	[33]
S32C. *pubescens*	Leaf	2	40	-	60	Esters: benzyl benzoate (50%), benzyl salicylate (23%); Monoterpenoids: p-cymene (12%)	[29]
S33*C. porrectum* (Roxb.) Kosterm	Leaf	1-2	90-94	5-9	-	Monoterpenoids: 1,8-cineole (34-36%), α-terpineol (19-21%), β-phellandrene (10-12%), 4-terpineol (6-9%), γ-terpinene (4-5%)	[34]
	Leaf	2	88-89	8-10	-	Monoterpenoids: linalool (78-88%), camphor (0-8%)	
	Leaf	1	92-99	0-4	-	Monoterpenoids: camphor (92-98%)	

	[S34]C. *rhyncophyllum*	Leaf	4	20	3	77	Esters: benzyl benzoate (70%); Monoterpenoids: β-phellandrene (10%)	[29]
		Bark	2	1	-	99	Phenylpropanoids: safrole (42%), methyl(*E*)-cinnamate (42%); Esters: benzyl benzoate (13%)	
	[S35]C. *scortechinii*	Leaf	4	30	4	66	Esters: benzyl benzoate (14%), benzyl salicylate (9%); Monoterpenoids: linalool (16%), β-phellandrene (17%)	
	[S36]*C. szech-uanense*	Leaf	1	88	1	8	Monoterpenoids: 1,8-cineole (43%), *α*-pinene (12%), camphene (8%), o-cymene (8%), bornyl acetate (7%)	[28]
	[S37]*C. subavenium*	Leaf	1	12	4	82	Phenylpropanoids: engenol (68%), engenol acetate (13%)	
	[S38]*C. tenuipile* Kosterm.	Leaf	-	-	-	-	Sesquiterpenoids: farnesol (58%), β-cubebene (9%), *α*-copaene (9%)	[35]
		Leaf	-	-	-	-	Monoterpenoids: geraniol (88%)	
		Leaf	-	-	-	-	Monoterpenoids: linalool (89%)	
	[S39]C. *zeylanicum*	Leaf	6	10	1	89	Phenylpropanoids: eugenol (90%)	[29]
		Bark	2	5	8	87	Sesquiterpenoids: β-caryophyllene (7%); Phenylpropanoids: cinnamaldehyde (44%)	
		Bark	1	9	-	90	Phenylpropanoids: (*E*)-cinnamaldehyde (69%); Esters: (*E*)-cinnamyl acetate (7%); Others: benzaldehyde (10%),	[36]
Sassafras	[S40]*S. albidum*	Leaf	<1	49-75	17-46	3-10	Monoterpenoids: geranial (11-27%), neral (10-18%), limonene (6-16%), *α*-pinene (3-12%); Sesquiterpenoids: (*E*)-caryophyllene (5-13%)	[37]
Ocotea	[S41]*O. bicolor* Vattimo-Gil	Leaf	3	-	49	33	Sesquiterpenoids: δ-cadinene (7%), β-sesquiphellandrene (7%), β-elemene (5%), *α*-cadinol (5%)	[38]
	[S42]*O. brenesii* Standl. [Syn: *Nectandra brenesii*	Leaf	<1	-	83	2	Sesquiterpenoids: *α*-copaene (21%), δ-cadinene (9%), β-caryophyllene (5%); spathulenol (7%), globulol (6%)	[39]
	(Standl.) C. K. Allen]	Wood	<1	2	67	1	Sesquiterpenoids: *α*-copaene (7%), caryophyllene oxide (6%), β-caryophyllene (6%), globulol (5%)	

		[S43]*O. caudata* (Nees) Mez	Leaf	<1	2	91	-	Sesquiterpenoids: germacrene D (56%), bicyclogermacrene (8%)	[40]
		[S44]*O. nigrescens* Vicentini	Leaf	<1	25	75	-	Monoterpenoids: α-pinene (7%), β-pinene (7%), linalool (6%); Sesquiterpenoids:β-caryophyllene (38%), α-copaene (6%)	[41]
		[S45]*O. puchury-major* Mart	Leaf	2	53	-	43	Monoterpenoids: eucaliptol (28%), sabinene (9%), α-terpineol (8%); Phenylpropanoids: safrole (39%)	[42]
		[S46]*O. splendens* (Meisn.)	Leaf	<1	1	93	7	Sesquiterpenoids: β-caryophyllene (51%), caryophyllene oxide (10%), α-humulene (6%); Others: linalool isovalerate (6%)	[41]
	Aniba	[S47]*A. canelilla* (Kunth) Mez	Leaf	1-2	2-9	3-20	71-88	Sesquiterpenoids: *E*-caryophyllene (0–7%), caryophyllene oxide (1–6%); Benzenoids:1-nitro-2-phenylethane (1N2F) (68–85%)	[43]
			Twig	0-1	5-28	1-4	73-91	Monoterpenoids: linalool (5–20%); Benzenoids: 1-nitro-2-phenylethane (1N2F) (71–90%)	
		[S48]*A. duckei* Kostermans	Branch	2	100	-	-	Monoterpenoids: linalool (94%)	[44]
		[S49]*A. parviflora* (Meisn.) Mez	Leaf	2	36	63	-	Monoterpenoids: β-phellandrene (15%), linalool (14%); Sesquiterpenoids: γ-eudesmol (13%)	[45]
			Branch	1	21	79	-	Monoterpenoids: linalool (12%), β-phellandrene (7%);Sesquiterpenoids:γ-eudesmol (17%), β-caryophyllene (16%), bicyclogermacrene (6%)	
		[S50]*A. rosaeodora* Ducke	Leaf	3	100	-	-	Monoterpenoids: linalool (94%)	[46]
			Stem	1	86	1	-	Monoterpenoids: linalool (86%)	[47]
			Leaf	1	81	4	-	Monoterpenoids: linalool (81%)	
			Branch	2	84	1	-	Monoterpenoids: linalool (84%)	
Machilus-Persea clade	*Machilus*	[S51]*M. balansa* (Airy Shaw) F.N. Wei & S.C. Tang	Leaf	<1	11	86	1	Sesquiterpenoids: bicyclogermacrene (42%), (*E*)-caryophyllene (9%), (*E*)-nerolidol (9%)	[8]
		[S52]*M. grandifolia* S.K. Lee & F.N. Wei	Leaf	<1	10	73	1	Sesquiterpenoids: (*E*)-nerolidol (23%), globulol (10%), selin-11-en-4α-ol (7%)	

	S53 *M. japonica*	Leaf	3	82	17	1	Monoterpenoids: α-phellandrene (15%), α-pinene (13%), thymol (13%), α-pinene (8%), α-terpineol (7%), carvacrol (6%); Sesquiterpenoids: β-caryophyllene (5%)	[48]
	S54 *M. kusanoi* Hayata	Leaf	<1	27	48	25	Monoterpenoids: α-terpineol (16%); Sesquiterpenoids: β-caryophyllene (23%), β-eudesmol (17%); Aldehydes: n-dodecanal (14%), n-decanal (10%)	[49]
	S55 *M. pseudolongifolia*	Leaf	2	6	92	2	Sesquiterpenoids: β-eudesmol (27%), α-cadinol (21%), viridiflorene (9%), α-caryophyllene (5%)	[50]
	S56 *M. philippinensis*	Leaf	1	40	57	3	Monoterpenoids: α-pinene (12%), *cis*-ocimene (7%); Sesquiterpenoids: β-caryophyllene (14%), α-cadinol (7%), spathulenol (6%), (*E*)-nerolidol (5%)	[51]
	S57 *M. thunbergii*	Leaf	<1	11	58	31	Sesquiterpenoids: β-caryophyllene (16%), α-humulene (11%), β-eudesmol (11%); Aldehydes: n-decanal (27%)	[52]
Dehaasia	S58 *D. cuneata*	Leaf	<1	98	1	-	Monoterpenoids: α-pinene (49%), camphene (20%), β-pinene (16%), limonene (8%)	[53]
Persea	S59 *P. americana*	Leaf	<1	11	43	42	Sesquiterpenoids: β-caryophyllene (17%); Phenylpropanoids: methyl eugenol (31%), estragole (9%)	[54]
		Leaf	0-1	0-14	6-74	10-81	Sesquiterpenoids: caryophyllene (1.14-37%); Phenylpropanoids: estragol (0-37%), methyl eugenol (2-22%); Others: 2-(8Z,11*Z*)-8,11-heptadecadienyl-furan (0-67%)	[55]
	S60 *P. duthiei*	Leaf	-	39	49	6	Monoterpenoids: limonene (10%), α-pinene (10%), β-pinene (10%); Sesquiterpenoids: (*E*)-nerolidol (13%), *epi*-cubebol (6%), β-caryophyllene (6%)	[56]
		Fruit	-	3	66	21	Sesquiterpenoids: (*E*)-nerolidol (25%), β-eudesmol (11%), selin-11-en-4-α-ol (9%), (*Z*)-nerolidol (8%)	
		Flower	-	5	70	16	Sesquiterpenoids: (*E)*-nerolidol (15%), *epi*-cubebol (12%), γ-	

								muurolene (12%), β-caryophyllene (8%)	
		[S61]*P. indica* (L.) Spreng.	Leaf	<1	1	23	76	Sesquiterpenoids: (*E*)-avocadodienofuran (15%), (*E*) avocadenynofuran (13%), β-caryophyllene (11%)	[57]
	Alseodaphne	[S62]*A. perakensis* (Gamble) Kosterm	Leaf	<1	-	91	-	Sesquiterpenoids: bicyclogermacrene(16%), δ-cadinene (12%), γ-cadinene (6%), aromadendrene (6%)	[58]
		[S63]*A. semecarpifolia*	Leaf	<1	7	70	9	Sesquiterpenoids: (*E*)-caryophyllene (32%), (*E*)-nerolidol (10%), germacrene D (7%), β-humulene (6%), caryophyllene oxide (6%), δ-cadinene (5%)	[59]
			Fruit	<1	38	36	1	Monoterpenoids: (*E*)-β-ocimene (15%); Sesquiterpenoids: *epi*-α-cadinol (9%), δ-cadinene (9%)	
		[S64]*A. velutina* Chev.	Leaf	-	19	70	-	Sesquiterpenoids: β-patchoulene (26%), β-caryophyllene (13%), aromadendrene (5%), (*Z*)-γ-bisabolene (7%)	[60]
	Phoebe	[S65]*P. angustifolia* Meisn.	Leaf	<1	69	30	-	Monoterpenoids: α-pinene (27%), β-pinene (21%), p-cymene (5%); Sesquiterpenoids: spathulenol (5%), (*E*)-caryophyllene (5%)	[8]
		[S66]*P. bournei* (Hemsl.)	Trunk xylem	2	24	38	28	Sesquiterpenoids: verbenone (8%), agaruspirol (18%), guaiol (11%), β-eudesmol (5%)	[61]
			Wood	0-1	-	39	61	Sesquiterpenoids: agarospirol (37%), eudesmol (31%), longiverbenone (7%)	[62]
			Wood		1	41	57	Sesquiterpenoids: agarospirol (37%), eudesmol (24%), guaiol (6%), uncineol (6%), elemol (6%)	
			Wood		0	33	41	Sesquiterpenoids: agarospirol (31%), eudesmol (25%), uncineol (6%)	
Caryodaphnopsis clade	*Caryodaphnopsis*	[S67]*C. tonkinensis* (Lecomte)	Leaf	1	72	25	1	Monoterpenoids: α-pinene (27%), β-pinene (23%); Sesquiterpenoids: bicyclogermacrene (9%)	[53]

Neocinnamomum clade	*Neocinnamomum*	[S68]*N. caudatum*	Bark	1	51.93	47	-	Monoterpenoids: β-pinene (13%), α-pinene (11%), Myrcene (8%), α-terpineol (7%); Sesquiterpenoids: α-cadinol (11%), γ-curcumene (6%)	[63]
			Leaf	1	84	11	-	Monoterpenoids: β-pinene (45%), myrcene (10%), α-pinene (9%)	
Cassytha clade	*Cassytha*	[S69]*C. filiformis* L.	Aerial parts	<1	-	84	-	Sesquiterpenoids: β-caryophyllene (5%). bicyclogermacrene (29%), spathulenol (35%)	[64]
			Aerial parts	<1	3	83	-	Sesquiterpenoids: β-elemene (5%), germacrene D (17%), bicyclogermacrene (26%), spathulenol (11%),	[65]
			Aerial parts		2	85	-	Sesquiterpenoids: germacrene D (16%), bicyclogermacrene (24%), spathulenol (19%)	
			Aerial parts		5	89	-	Sesquiterpenoids: α-copaene (8%), β-caryophyllene (31%), α-humulene (17%), germacrene D (7%), carryophyllene oxide (5%)	
			Aerial parts		3	89	-	Sesquiterpenoids: β-caryophyllene (35%), α-humulene (22%), germacrene D (8%)	
			Aerial parts		-	90	-	Sesquiterpenoids: β-caryophyllene (30%), α-humulene (18%), germacrene D (12%)	
		[S70]*C. pubescens*	Aerial parts	<1	1	79	-	Sesquiterpenoids: β-caryophyllene (8%), caryophyllene oxide (17%), spathulenol (32%)	[64]
			Aerial parts		1	94	-	Sesquiterpenoids: β-caryophyllene (31%), bicyclogermacrene (30%), spathulenol (11%)	
			Aerial parts		-	89	-	Sesquiterpenoids: β-caryophyllene (24%), bicyclogermacrene (8%), (*E*)-nerolidol (27%), spathulenol (6%)	
Beilschmiedia-	*Cryptocarya*	[S71]*C. amygdalina*	Bark	<1	3	96	-	Sesquiterpenoids: β-caryophyllene (35 %), α-copaene (14%), bicyclogermacrene (8%)	[66]
		[S72]*C. alba* (Molina) Looser	Aerial parts	1	58	7	1	Monoterpenoids: α-terpineol (25%), 1,8-cineole (22%), β-phellandrene (15%)	[67]

Cryptocarya clade	[S73]*C. aschersoniana*	Leaf	4	62	33	2	Monoterpenoids: limonene (42%), linalool (10%); Sesquiterpenoids: nerolidol (9%), spathulenol (7%)	[68]
	[S74]*C. botelhensis*	Leaf		56	18	27	Monoterpenoids: α-pinene (23%), β-pinene (9%), trans-verbenol (8%), *trans*-pinocarveol (6%), myrtenal (5%)	[69]
	[S75]*C. bellendenkerana*	Leaf	<1	20	10	1	Monoterpenoids: limonene (8%), β-phellandrene (12%); Sesquiterpenoids: viridiflorene (9%)	[70]
	[S76]*C. cocosoides*	Leaf	<1	2	42	19	Monoterpenoids: neral (5%), geranial (6%); Sesquiterpenoids: bicyclogermacrene (26%), spathulenol (17%); Aromatic esters: massoia lactone (15%)	[70]
	[S77]*C. concinna*	Leaf	<1	25	74	-	Monoterpenoids: α-pinene (8%), β-pinene (9%); Sesquiterpenoids: (*E*)-caryophyllene (12%), spathulenol (12%), caryophyllene oxide (20%)	[8]
		Leaf	<1	83	12	-	Monoterpenoids: α-pinene (27%), β-pinene (31%), myrcene (11%), (*E*)-β-ocimene (9%); Sesquiterpenoids: (*E*)-caryophyllene (5%)	
	[S78]*C. impressa*	Leaf	<1	-	91	1	Sesquiterpenoids: α-cadinol (41%), 1,10-di-*epi*-cubenol (13%), germacrene D (7%), γ-cadinene (6%)	[71]
	[S79]*C. impressa*	Leaf	<1	20	62	15	Sesquiterpenoids: bicyclogermacrene (19%), (*E*)-caryophyllene (11%), (*E, E*)-α-farnesene (8%), α-humulene (6%); Aldehydes: dodecanal: (11%)	[8]
	[S80]*C. infectoria*	Leaf	<1	-	91	-	Sesquiterpenoids: β-caryophyllene (25%), bicyclogermacrene (15%), germacrene D (10%), δ-cadinene (7%), globulol (5%)	[71]
		Leaf	<1	5	88	-	Sesquiterpenoids: germacrene D (56%), bicyclogermacrene (11%), δ-elemene (5%)	[8]
	[S81]*C. lividula*	Leaf	<1	12	47	7	Sesquiterpenoids: bicyclogermacrene (26%), spathulenol (21%), β-eudesmol (6%)	[70]
		Bark	1	2	1	96	Lactones: C-10 massoia lactone (68%), C-12 massoia lactone (17%)	[72]

	[S82]*C. massoy* (Oken) Kosterm.	Heart-wood	1	-	-	100	Lactones: C-10 massoia lactone (68%), C-12 massoia lactone (28%)	
	[S83]*C. moschata*	Leaf	<1	77	4	19	Monoterpenoids: linalool (34%), α-terpinene (17%), γ-terpinene (10%), 1,8-cineole (6%)	[69]
	[S87]*C. mandioccana* Meisner	Leaf		10	87	-	Sesquiterpenoids: β-caryophyllene (14%), spathulenol (10%), caryophyllene oxide (8%), δ-cadinene (7%)	[73]
		Leaf		3	62	35	Sesquiterpenoids: β-caryophyllene (14%), spathulenol (10%), caryophyllene oxide (8%), δ-cadinene (7%), bicyclogermacrene (6%)	[69]
	[S85]*C. rugulosa*	Leaf		-	83	-	Sesquiterpenoids: bicyclogermacrene (16%), δ-cadinene (14%), α-copaene (12%), aromadendrene (6%)	[71]
	[S86]*C. saligna*	Leaf		10	62	28	Sesquiterpenoids: germacrene D (16%), bicyclogermacrene (14%), spathulenol (12%), germacrene B (6%)	[69]
Beilschmiedia	[S87]*B. alloiophylla* (Rusby) Kosterm.	Leaf	<1	51	46	3	Monoterpenoids: cis-β-ocimene (19%), α-pinene (12%), trans-β-ocimene (9%); Sesquiterpenoids: bicyclogermacrene (9%), germacrene D (19%)	[74]
	[S88]*B. brenesii* C.K.	Leaf	<1	3	69	27	Sesquiterpenoids: germacrene D (19%), β-caryophyllene (13%), α-copaene (9%), α-humulene (8%), δ-cadinene (6%); ketone: 2-undecanone (13%); aldehyde: trans-2-hexenal (9%)	
	[S89]*B. costaricensis* Mez & Pittier	Leaf	<1	7	88	5	Sesquiterpenoids: α-bisabolol (72%); Other: *cis*-3-Hexenol (5%)	
	[S90]*B. erythrophloia*	Leaf	<1	36	63	-	Monoterpenoids: (*Z*)-β-ocimene (26%); Sesquiterpenoids: bicyclogermacrene (31%), (*E*)-caryophyllene (18%)	[8]
	[S91]*B. madang*	Leaf	<1	4	86	1	Sesquiterpenoids: δ-cadinene (17%), α-cubebene (11%), β-caryophyllene (10%), α-cadinol (6%),	[75]

						(*E*)-nerolidol (5%), bicyclogermacrene (7%)	
	Bark	<1	3	79	-	Sesquiterpenoids: δ-cadinene (21%), α-cubebene (6%), α-cadinol (11%), β-caryophyllene (7%)	
[S92]*B. pendula* (Sw.) Hemsl.	Leaf	<1	30	49	5	Monoterpenoids: β-pinene (10%), α-pinene (8%); Sesquiterpenoids: bicyclogermacrene (7%), β-caryophyllene (9%)	[76]
	Branch	<1	11	84	2	Sesquiterpenoids: β-caryophyllene (17%), β-selinene (9%), bicyclogermacrene (9%), α-cadinol (6%)	
[S93]*B. robusta*	Leaf	<1	11	88	-	Sesquiterpenoids: (*E*)-caryophyllene (23%), α-humulene (13%), bicyclogermacrene (9%), germacrene D (20%)	[8]
[S94]*B. tilaranensis* Sa. Nashida	Leaf	<1	5	94	1	Sesquiterpenoids: germacrene D (55%), β-caryophyllene (15%), δ-cadinene (5%)	[74]
[S95]*B. yunnanensis*	Leaf	<1	24	75	-	Sesquiterpenoids: (*E*)-caryophyllene (16%), α-humulene (10%), bicyclogermacrene (8%), 9-epi-(*E*)-caryophyllene (21%)	[8]

S1-S95: The numbers we assigned to each different species.

Table S2. The overview of TPSs related to terpenoid biosynthesis in Lauraceae.

Classification	Gene	GenBank login number	Plant origin	Substrate	Product	Functional analysis method	Reference
TPS-a	*LnTPS2*	KR336615	*Laurus nobilis*	FPP	δ-Cadinene and γ-cadinene	*Escherichia coli*	[77]
	CbTPS4	MW196674	*Cinnamomum burmannii*	GPP/FPP	Limonene, myrcene and linalool/α-copaene and γ-muurolene		[78]
	CbTPS5	MW196675	*Cinnamomum burmannii*	GPP/FPP	Myrcene/caryophyllene and humulene		[78]
	CbTPS6	MW196676	*Cinnamomum burmannii*	GPP/FPP	Myrcene, linalool, limonene, and terpineol/Cadina-1(10),4-dine and α-copaene		[78]
TPS-b	*CtGES*	AJ457070	*Cinnamomum tenuipilum*	GPP	Geraniol	*Escherichia coli*	[35]
	LcuTPS22	—	*Litsea cubeba*	GPP	α-Pinene, β-pinene, camphene, 1,8-cineole and camphor	*Escherichia coli*	[79]
	CcTPS16	OM721572	*Cinnamomum camphora*	GPP	1,8-Cineole	*Escherichia coli*	[80]
	LnTPS1	KR336614.1	*Laurus nobilis*	GPP	1,8-Cineole	*Escherichia coli*	[77]
	CbTPS2	MW196672	*Cinnamomum burmannii*	GPP	Linalool, α-pinene and β-pinene	*Escherichia coli*	[78]
	CbTPS3	MW196673	*Cinnamomum burmannii*	GPP	Linalool	*Escherichia coli*	[78]
	CbTPS7	MW196677	*Cinnamomum burmannii*	GPP/FPP	Linalool/ nerolidol	*Escherichia coli*	[78]
	LcTPS1	HQ651178	*Litsea cubeba.*	GPP	*Trans*-ocimene	*Escherichia coli*	[81]
	LcTPS2	HQ651179	*Litsea cubeba*	GPP	α-Thujene	*Escherichia coli*	[81]
	LcTPS3	HQ651180	*Litsea cubeba*	GPP	α-Thujene and (+)-sabinene	*Escherichia coli*	[81]
	CbTPS1	MW196671	*Cinnamomum burmanni*	GPP	(+)-Borneol	*Escherichia coli*	[78]
TPS-e/f	*LnTPS3*	KR336616	*Laurus nobilis*	GGPP/eeFPP	Geranyllinalool/*trans*-nerolido	*Escherichia coli*	[77]
TPS-g	*LcuTPS42*	—	*Litsea cubeba*	GPP	Phellandrene, linalool and geraniol	*Escherichia coli*	[79]
	CcTPS54	OM721573	*Cinnamomum camphora*	GPP/FPP	Linalool/(*iso*)nerolidol	*Escherichia coli*	[80]
Ohers	*CoLIS-LL*<br>*CoLIS-B1*<br>*CoLIS-D4*	JX028232<br>JX133145<br>JX028230	*Cinnamomum osmophloeum*	GPP/FPP	Linalool/*trans*-nerolidol	*Escherichia coli*	[82]

CoLIS-E1	JX133146
CoLIS-HS	JX028231
CoLIS-L	JX133147
CoLIS-M21	JX133148
CoLIS-S1	JX133149
CoLIS-T1	JX028233

References

1. Wang, H.; Liu, Y. Chemical Composition and Antibacterial Activity of Essential Oils from Different Parts of *Litsea Cubeba*. *C&B* **2010**, *7*, 229–235, doi:10.1002/cbdv.200800349.
2. Wang, X.-Y.; Li, B.-T.; Wen, Z.-Q. Volatile Constituents of the Leaf and Fruit Essential Oils of *Litsea Cubeba* (Lour.) Pers. Growing Wild in Baoshan Region, China. *Nat. Prod. Res.* **2025**, *39*, 241–247, doi:10.1080/14786419.2023.2261137.
3. Ho, C.; Ou, O.; Liu, Y.; Hung, C.; Tsai, M.; Liao, P.; Wang, E.Ic.; Chen, Y.; Su, Y. Compositions and *in Vitro* Anticancer Activities of the Leaf and Fruit Oils of *Litsea Cubeba* from Taiwan. *Natural Product Communications* **2010**, *5*, 1934578X1000500, doi:10.1177/1934578X1000500425.
4. Si, L.; Chen, Y.; Han, X.; Zhan, Z.; Tian, S.; Cui, Q.; Wang, Y. Chemical Composition of Essential Oils of *Litsea Cubeba* Harvested from Its Distribution Areas in China. *Molecules* **2012**, *17*, 7057–7066, doi:10.3390/molecules17067057.
5. Son, L.C.; Dai, D.N.; Thang, T.D.; Huyen, D.D.; Ogunwande, I.A. Analysis of the Essential Oils from Five Vietnamese *Litsea* Species (Lauraceae). *J. Essent. Oil Bear. Plants* **2014**, *17*, 960–971, doi:10.1080/0972060X.2014.935068.
6. Ran, H.; Feng, L.; Mao, Y.; Zhou, L.; Liu, S. Identification and analysis of volatile components in essential oil from four Lauraceae wild species leaves in Chongqing by GC-MS. *Scientia silvae sinicae* **2018**, *54*, 91–103, doi:10．11707 / j．1001-7488．20180710.
7. Guzmán-Gutiérrez, S.L.; Gómez-Cansino, R.; García-Zebadúa, J.C.; Jiménez-Pérez, N.C.; Reyes-Chilpa, R. Antidepressant Activity of *Litsea Glaucescens* Essential Oil: Identification of β-Pinene and Linalool as Active Principles. *Journal of Ethnopharmacology* **2012**, *143*, 673–679, doi:10.1016/j.jep.2012.07.026.
8. Chau, D.T.M.; Chung, N.T.; Huong, L.T.; Hung, N.H.; Ogunwande, I.A.; Dai, D.N.; Setzer, W.N. Chemical Compositions, Mosquito Larvicidal and Antimicrobial Activities of Leaf Essential Oils of Eleven Species of Lauraceae from Vietnam. *Plants (Basel)* **2020**, *9*, 606, doi:10.3390/plants9050606.
9. Linh, L.D.; Ban, P.H.; Hoi, T.M.; Huong, L.T.; Ogunwande, I.A. Compositions of Essential Oils from the Leaf, Stem and Fruit of *Neolitsea Buisanensis* (Lauraceae) from Vietnam. *J. Essent. Oil Bear. Plants* **2018**, *21*, 1257–1265, doi:10.1080/0972060X.2018.1527729.
10. Jani, N.A.; Sirat, H.M.; Ahmad, F.; Ali, N.A.M.; Zainal, M.H. Chemical Composition, Antibacterial and α-Glucosidase Inhibitory Activities of the Essential Oils of *Neolitsea Coccinea* (Lauraceae). *Natural Product Communications* **2016**, *11*, 1934578X1601101231, doi:10.1177/1934578X1601101231.

11. Shyu, J.-G.; Hsu, C.-K.; Hsu, K.-P.; Yang, M.-L.; Wei, L.Y.; Ho, H.-T.; Ho, C.-L. Chemical Composition, *in Vitro* Antibacterial and Antifungal Activities of Different Parts Essential Oils of *Neolitsea Sericea* Var. *Aurata* From Taiwan. *Nat. Prod. Commun.* **2023**, *18*, 1934578X231166290, doi:10.1177/1934578X231166290.
12. Liu, Y.; Wang, H.; Wei, S.; Cai, X. Characterisation of the Essential Oil from Different Aerial Parts of *Lindera Chunii* Merr. (Lauraceae). *Natural Product Research* **2013**, *27*, 1804–1807, doi:10.1080/14786419.2012.761619.
13. Chen, F.; Miao, X.; Lin, Z.; Xiu, Y.; Shi, L.; Zhang, Q.; Liang, D.; Lin, S.; He, B. Disruption of Metabolic Function and Redox Homeostasis as Antibacterial Mechanism of *Lindera Glauca* Fruit Essential Oil against Shigella Flexneri. *Food Control* **2021**, *130*, 108282, doi:10.1016/j.foodcont.2021.108282.
14. Sun, Z.; Su, X.; Lin, Y.; Long, C.; Zhang, Y.; Zhao, T. Chemical Composition, and Antioxidant and Cholinesterase Inhibitory Activities of <i>Lindera Glauca<\i> Fruit Essential Oil and Molecular Docking Studies of Six Selected Compounds. *Horticulturae* **2023**, *9*, 289, doi:10.3390/horticulturae9020289.
15. Liu, Z.; Chen, H. GC-MS Analysis of Essential Oil from the Bark of *Lindera Obtusiloba*. *Chem Nat Compd* **2012**, *48*, 696–697, doi:10.1007/s10600-012-0354-6.
16. Joshi, S.C.; Verma, A.R.; Mathela, C.S. Antioxidant and Antibacterial Activities of the Leaf Essential Oils of Himalayan Lauraceae Species. *Food and Chemical Toxicology* **2010**, *48*, 37–40, doi:10.1016/j.fct.2009.09.011.
17. Dai, D.N.; Thang, T.D.; Pino, J.A. Essential Oil of *Lindera Rufa* Hook. f. Leaves from Vietnam. *Journal of Essential Oil Bearing Plants* **2013**, *16*, 832–834, doi:10.1080/0972060X.2013.813269.
18. Wei, G.; Chen, H.; Kong, L.; Li, X.; Ma, C.; Jiang, H. Composition and Bioactivity of the Essential Oil from the Leaves of *Lindera Setchuenensis*. *Chem Nat Compd* **2016**, *52*, 520–522, doi:10.1007/s10600-016-1696-2.
19. Yan, R.; Yang, Y.; Zou, G. Cytotoxic and Apoptotic Effects of *Lindera Strychnifolia* Leaf Essential Oil. *J. Essent. Oil Res.* **2014**, *26*, 308–314, doi:10.1080/10412905.2013.840811.
20. Yan, R.; Yang, Y.; Zeng, Y.; Zou, G. Cytotoxicity and Antibacterial Activity of Lindera Strychnifolia Essential Oils and Extracts. *J. Ethnopharmacol.* **2009**, *121*, 451–455, doi:10.1016/j.jep.2008.06.010.
21. Mediouni Ben Jemâa, J.; Tersim, N.; Toudert, K.T.; Khouja, M.L. Insecticidal Activities of Essential Oils from Leaves of *Laurus Nobilis* L. from Tunisia, Algeria and Morocco, and Comparative Chemical Composition. *Journal of Stored Products Research* **2012**, *48*, 97–104, doi:10.1016/j.jspr.2011.10.003.
22. Caputo, L.; Nazzaro, F.; Souza, L.F.; Aliberti, L.; De Martino, L.; Fratianni, F.; Coppola, R.; De Feo, V. *Laurus Nobilis*: Composition of Essential Oil and Its Biological Activities. *Molecules* **2017**, *22*, 930, doi:10.3390/molecules22060930.
23. Stefanova, G.; Girova, T.; Gochev, V.; Stoyanova, M.; Petkova, Z.; Stoyanova, A.; Zheljazkov, V.D. Comparative Study on the Chemical Composition of Laurel (*Laurus Nobilis* L.) Leaves from Greece and Georgia and the Antibacterial Activity of Their Essential Oil. *Heliyon* **2020**, *6*, e05491, doi:10.1016/j.heliyon.2020.e05491.
24. Liu, X.; Zhou, S.; Huang, Y.; Chen, M.; Wang, W.; Wang, J.; Hao, E.; Wu, H.; Li, Y. Chemical Composition, Antioxidant Activity, and Anti-Bacterial Activity of Essential

Oils from Different Organs of *Cinnamomum Burmanni*. *Journal of Essential Oil Bearing Plants* **2023**, *26*, 787–801, doi:10.1080/0972060X.2023.2239847.

25. Wang, Y.; Zhang, L.-T.; Feng, Y.-X.; Zhang, D.; Guo, S.-S.; Pang, X.; Geng, Z.-F.; Xi, C.; Du, S.-S. Comparative Evaluation of the Chemical Composition and Bioactivities of Essential Oils from Four Spice Plants (Lauraceae) against Stored-Product Insects. *Industrial Crops and Products* **2019**, *140*, 111640, doi:10.1016/j.indcrop.2019.111640.
26. Hu, W.; Jiang, X.; Yang, H.; Zhang, T.; Zhao, L.; Xiao, F. Components and their contents in essential oils from different parts of camphor type. *Journal of Northwest A&F University (Nat. Sci. Ed.)* **2014**, *42*, 126–132, doi:10.13207/j.cnki.jnwafu.2014.10.052.
27. Poudel, D.K.; Rokaya, A.; Ojha, P.K.; Timsina, S.; Satyal, R.; Dosoky, N.S.; Satyal, P.; Setzer, W.N. The Chemical Profiling of Essential Oils from Different Tissues of *Cinnamomum Camphora* L. and Their Antimicrobial Activities. *Molecules* **2021**, *26*, 5132, doi:10.3390/molecules26175132.
28. Zhang, J.; Huang, T.; Zhang, J.; Shi, Z.; He, Z. Chemical Composition of Leaf Essential Oils of Four *Cinnamomum* Species and Their Larvicidal Activity against *Anophelus Sinensis* (Diptera: Culicidae). *Journal of Essential Oil Bearing Plants* **2018**, *21*, 1284–1294, doi:10.1080/0972060X.2018.1552205.
29. Jantan, I.B.; Karim Moharam, B.A.; Santhanam, J.; Jamal, J.A. Correlation between Chemical Composition and Antifungal Activity of the Essential Oils of Eight *Cinnamomum* . Species. *Pharmaceutical Biology* **2008**, *46*, 406–412, doi:10.1080/13880200802055859.
30. Jeyaratnam, N.; Nour, A.H.; Kanthasamy, R.; Nour, A.H.; Yuvaraj, A.R.; Akindoyo, J.O. Essential Oil from *Cinnamomum Cassia* Bark through Hydrodistillation and Advanced Microwave Assisted Hydrodistillation. *Industrial Crops and Products* **2016**, *92*, 57–66, doi:10.1016/j.indcrop.2016.07.049.
31. Chen, G.; Sun, F.; Wang, S.; Wang, W.; Dong, J.; Gao, F. Enhanced Extraction of Essential Oil from Cinnamomum Cassia Bark by Ultrasound Assisted Hydrodistillation. *Chinese Journal of Chemical Engineering* **2021**, *36*, 38–46, doi:10.1016/j.cjche.2020.08.007.
32. Yang, H.; Zhang, T.; Wang, X.; Wen, S.; Guo, Y.; Jiang, X. A Study on the Chemical Components in Essential Oil from Leaves of *Cinnamomum Kanehirae* and Chemotype Divisions. *Acta Agriculturae Universitatis Jiangxiensis* **2016**, *38*, 668–673, doi:10.13836/j.jjau.2016095.
33. Lee, S.-C.; Xu, W.-X.; Lin, L.-Y.; Yang, J.-J.; Liu, C.-T. Chemical Composition and Hypoglycemic and Pancreas-Protective Effect of Leaf Essential Oil from Indigenous Cinnamon (Cinnamomum Osmophloeum Kanehira). *J. Agric. Food Chem.* **2013**, *61*, 4905–4913, doi:10.1021/jf401039z.
34. Qiu, F.; Wang, X.; Zheng, Y.; Wang, H.; Liu, X.; Su, X. Full-Length Transcriptome Sequencing and Different Chemotype Expression Profile Analysis of Genes Related to Monoterpenoid Biosynthesis in *Cinnamomum Porrectum*. *International Journal of Molecular Sciences* **2019**, *20*, 6230, doi:10.3390/ijms20246230.
35. Yang, T.; Li, J.; Wang, H.; Zeng, Y. A Geraniol-Synthase Gene from *Cinnamomum Tenuipilum*. *Phytochemistry* **2005**, *66*, 285–293, doi:10.1016/j.phytochem.2004.12.004.

36. Unlu, M.; Ergene, E.; Unlu, G.V.; Zeytinoglu, H.S.; Vural, N. Composition, Antimicrobial Activity and in Vitro Cytotoxicity of Essential Oil from Cinnamomum Zeylanicum Blume (Lauraceae). *Food Chem. Toxicol.* **2010**, *48*, 3274–3280, doi:10.1016/j.fct.2010.09.001.
37. Kaler, K.M.; Setzer, W.N. Seasonal Variation in the Leaf Essential Oil Composition of *Sassafras Albidum*. *Natural Product Communications* **2008**, *3*, 1934578X0800300, doi:10.1177/1934578X0800300529.
38. Damasceno, C.S.B.; Oliveira, L.F.D.; Szabo, E.M.; Souza, Â.M.; Dias, J.F.G.; Miguel, M.D.; Miguel, O.G. Chemical Composition, Antioxidant and Biological Activity of Ocotea Bicolor Vattimo-Gil (LAURACEAE) Essential Oil. *Braz. J. Pharm. Sci.* **2018**, *53*, doi:10.1590/s2175-97902017000417298.
39. Chaverri, C.; Cicció, J.F. Essential Oil of Trees of the Genus *Ocotea* (Lauraceae) in Costa Rica. I. *Ocotea Brenesii*. *Rev. Biol. Trop.* **2014**, *53*, 431, doi:10.15517/rbt.v53i3-4.14611.
40. Gil, E.; Cuca, L.E.; Delgado, W.A. Chemical Composition and Antimicrobial Activity of the Essential Oil of the Leaves of Ocotea Caudata (Nees) Mez (Lauraceae) from Colombia.
41. Yamaguchi, K.K.D.L.; Alcantara, J.M.; Lima, E.S.; Veiga-Junior, V.F.D. Chemical Composition and Platelet Aggregation Activity of Essential Oils of Two Species of the Genus *Ocotea* (Lauraceae). *J. Essent. Oil Bear. Plants* **2013**, *16*, 518–523, doi:10.1080/0972060X.2013.855364.
42. Leporatti, M.L.; Pintore, G.; Foddai, M.; Chessa, M.; Piana, A.; Petretto, G.L.; Masia, M.D.; Mangano, G.; Nicoletti, M. Chemical, Biological, Morphoanatomical and Antimicrobial Study of *Ocotea Puchury-Major* Mart. *Nat. Prod. Res.* **2014**, *28*, 294–300, doi:10.1080/14786419.2013.858338.
43. Cruz, E.D.N.S.D.; Barros, L.D.S.P.; Guimarães, B.D.A.; Mourão, R.H.V.; Maia, J.G.S.; Setzer, W.N.; Da Silva, J.K.D.R.; Figueiredo, P.L.B. Seasonal Variation in Essential Oil Composition and Antioxidant Capacity of Aniba Canelilla (Lauraceae): A Reliable Source of 1-Nitro-2-Phenylethane. *Molecules* **2023**, *28*, 7573, doi:10.3390/molecules28227573.
44. Barros Gomes, P.R.; Oliveira Cunha, M.J.; De Sousa, D.A.; Reis, J.B.; Melo, A.V.; De Freitas, A.C.; Lima Hunaldo, V.K.; Fontenele, M.A.; De Paula, M.D.L.; Louzeiro, H.C.; et al. Chemical Study and Antifungal Activity of the Essential Oil of the Branches of *Aniba Duckei* Kostermans. *Journal of Essential Oil Bearing Plants* **2019**, *22*, 1554–1561, doi:10.1080/0972060X.2019.1700829.
45. Da Silva, J.K.R.; Maia, J.G.S.; Dosoky, N.S.; Setzer, W.N. Antioxidant, Antimicrobial, and Cytotoxic Properties of *Aniba Parviflora* Essential Oils from the Amazon. *Nat. Prod. Commun.* **2016**, *11*, 1934578X1601100738, doi:10.1177/1934578X1601100738.
46. Teles, A.M.; Silva-Silva, J.V.; Fernandes, J.M.P.; Calabrese, K.D.S.; Abreu-Silva, A.L.; Marinho, S.C.; Mouchrek, A.N.; Filho, V.E.M.; Almeida-Souza, F. Aniba Rosaeodora (Var. Amazonica Ducke) Essential Oil: Chemical Composition, Antibacterial, Antioxidant and Antitrypanosomal Activity. *Antibiotics* **2020**, *10*, 24, doi:10.3390/antibiotics10010024.
47. Krainovic, P.M.; Almeida, D.R.A.D.; Veiga Junior, V.F.D.; Sampaio, P.D.T.B. Changes in Rosewood (Aniba Rosaeodora Ducke) Essential Oil in Response to Management of Commercial Plantations in Central Amazonia. *For. Ecol. Manage.* **2018**, *429*, 143–157, doi:10.1016/j.foreco.2018.07.015.

48. Ho, C.; Su, Y. Composition, Antioxidant and Antimicrobial Activities of the Leaf Essential Oil of *Machilus Japonica* from Taiwan. *Natural Product Communications* **2012**, *7*, 1934578X1200700, doi:10.1177/1934578X1200700136.
49. Ho, C.; Hsu, K.; Tseng, Y.; Wang, E.I.C.; Liao, P.; Chou, J.-C.; Lin, C.-N.; Su, Y.-C. Composition and Antimicrobial Activities of the Leaf Essential Oil of *Machilus Kusanoi* from Taiwan. *Natural Product Communications* **2011**, *6*, 1934578X1100600, doi:10.1177/1934578X1100600228.
50. Ho, C.; Liao, P.; Hsu, K.; Wang, E.Ic.; Dong, W.; Su, Y. Composition and Antimicrobial and Anti-Wood-Decay Fungal Activities of the Leaf Essential Oils of *Machilus Pseudolongifolia* from Taiwan. *Natural Product Communications* **2010**, *5*, 1934578X1000500, doi:10.1177/1934578X1000500735.
51. Ho, C.; Hsu, K.; Wang, E.Ic.; Lin, C.; Su, Y. Composition and Anti-Wood-Decay Fungal Activities of the Leaf Essential Oil of *Machilus Philippinensis* from Taiwan. *Natural Product Communications* **2010**, *5*, 1934578X1000500, doi:10.1177/1934578X1000500233.
52. Su, Y.C.; Hsu, K.P.; Li, S.C.; Ho, C.L. Composition, *in Vitro* Cytotoxicity, and Anti-Mildew Activities of the Leaf Essential Oil of *Machilus Thunbergii* from Taiwan. *Natural Product Communications* **2015**, *10*, 1934578X1501001, doi:10.1177/1934578X1501001153.
53. Ogunwande, I.A.; Huong, L.T.; Chau, D.T.M.; Dai, D.N. Essential Oils of Lauraceae: Constituents and Antimicrobial Activity of *Dehaasia Cuneata* (Blume) Blume and *Caryodaphnopsis Tonkinensis* (Lecomte) Airy-Shaw from Vietnam. *Rec. Nat. Prod.* **2022**, 477–482, doi:10.25135/rnp.301.2110.2240.
54. Larijani, K.; Rustaiyan, A.; Abroomand Azar, P.; Nematollahi, F.; Taban, S. Composition of Essential Oil of Leaves of *Persea Americana* Cultivated in Iran. *Chem Nat Compd* **2010**, *46*, 489–490, doi:10.1007/s10600-010-9655-9.
55. Nasri, C.; Halabi, Y.; Aghzaf, S.; Nounah, I.; Brunel, M.; Oubihi, A.; El-Guorrami, O.; Harhar, H.; Costa, J.; Tabyaoui, M. Seven *Persea Americana* Varieties Essential Oils Comparison: Chemical Composition, Toxicity, Antibacterial, and Antioxidant Activities. *Biocatalysis and Agricultural Biotechnology* **2022**, *44*, 102468, doi:10.1016/j.bcab.2022.102468.
56. Padalia, R.C.; Joshi, S.C.; Bisht, D.S.; Mathela, C.S. Essential Oil Composition of *Persea Duthiei*. *Chem Nat Compd* **2009**, *45*, 745–747, doi:10.1007/s10600-009-9418-7.
57. Pino, J.A.; Fernandes, P.; Marbot, R.; Rosado, A.; Fontinha, S.S. Leaf Oils of *Helichrysum Melaleucum* Rchb. Ex Holl., *Oenanthe Divaricata* (R. Br.) Mabb. and *Persea Indica* (L.) Spreng. from Madeira. *Journal of Essential Oil Research* **2004**, *16*, 487–489, doi:10.1080/10412905.2004.9698778.
58. Anuar, M.Z.A.; Salleh, W.M.N.H.W.; Khamis, S.; Nafiah, M.A.; Mat Said, Z. Essential Oil Composition of *Alseodaphne Perakensis* (Gamble) Kosterm from Malaysia. *Natural Product Research* **2021**, *35*, 508–511, doi:10.1080/14786419.2019.1636245.
59. Verma, R.S.; Kumar, A.; Mishra, P.; Kuppusamy, B.; Padalia, R.C.; Sundaresan, V. Essential Oil Constituents of *Alseodaphne Semecarpifolia* from Central Western Ghats, India. *Chem Nat Compd* **2016**, *52*, 516–517, doi:10.1007/s10600-016-1694-4.
60. Cuong, N.T.; Ban, P.H.; Chung, M.V. Chemical Composition and Antioxidant Activity of the Essential Oil of *Alseodaphne Velutina* Chev. from Viet Nam. *Natural Product Research* **2022**, *36*, 617–620, doi:10.1080/14786419.2020.1789633.

61. Zhang, Q.; Ning, L.; Ding, W.; Xing, H.; Zhou, Q.; Wei, Z. Chemical Constituents of the Essential Oil Extracted from Phoebe Bournei. *Chem. Nat. Compd.* **2019**, *55*, 754–755, doi:10.1007/s10600-019-02801-9.
62. Yang, H.; Zhang, S.; Gu, Y.; Peng, J.; Huang, X.; Guo, H.; Chen, L.; Jiang, Y.; Liu, M.; Luo, X.; et al. Identification and Variation Analysis of the Composition and Content of Essential Oil and Fragrance Compounds in Phoebe Zhennan Wood at Different Tree Ages. *Front. Plant Sci.* **2024**, *15*, doi:10.3389/fpls.2024.1368894.
63. Jena, S.; Ray, A.; Mohanta, O.; Das, P.K.; Sahoo, A.; Nayak, S.; Panda, P.C. *Neocinnamomum Caudatum* Essential Oil Ameliorates Lipopolysaccharide-Induced Inflammation and Oxidative Stress in RAW 264.7 Cells by Inhibiting NF-κB Activation and ROS Production. *Molecules* **2022**, *27*, 8193, doi:10.3390/molecules27238193.
64. Brophy, J.J.; Goldsack, R.J.; Forster, P.I. The Essential Oils of Some Australian *Cassytha* Species (Lauraceae). *Journal of Essential Oil Research* **2009**, *21*, 543–546, doi:10.1080/10412905.2009.9700239.
65. Ouattara, Z.A.; Sangaré, N.; Mamyrbekova-Bekro, A.J.; Békro, Y.-A.; Tomi, P.; Paoli, M.; Bighelli, A.; Tomi, F. Composition and Chemical Variability of Essential Oils Isolated from Aerial Parts of *Cassytha Filiformis* from Côte d'Ivoire. *Natural Product Communications* **2018**, *13*, 1934578X1801300225, doi:10.1177/1934578X1801300225.
66. Ray, A.; Jena, S.; Sahoo, A.; Kamila, P.K.; Das, P.K.; Mohanty, S.; Nayak, S.; Panda, P.C. Chemical Composition, Antioxidant, Anti-Inflammatory and Anticancer Activities of Bark Essential Oil of *Cryptocarya Amygdalina* from India. *Journal of Essential Oil Bearing Plants* **2021**, *24*, 617–631, doi:10.1080/0972060X.2021.1950051.
67. Touma, J.; Navarro, M.; Sepúlveda, B.; Pavon, A.; Corsini, G.; Fernández, K.; Quezada, C.; Torres, A.; Larrazabal-Fuentes, M.J.; Paredes, A.; et al. The Chemical Compositions of Essential Oils Derived from *Cryptocarya Alba* and *Laurelia Sempervirens* Possess Antioxidant, Antibacterial and Antitumoral Activity Potential. *Molecules* **2020**, *25*, 5600, doi:10.3390/molecules25235600.
68. Andrade, P.M.D.; Melo, D.C.D.; Alcoba, A.E.T.; Ferreira Júnior, W.G.; Pagotti, M.C.; Magalhães, L.G.; Santos, T.C.L.D.; Crotti, A.E.M.; Alves, C.C.F.; Miranda, M.L.D. Chemical Composition and Evaluation of Antileishmanial and Cytotoxic Activities of the Essential Oil from Leaves of *Cryptocarya Aschersoniana* Mez. (Lauraceae Juss.). *An. Acad. Bras. Ciênc.* **2018**, *90*, 2671–2678, doi:10.1590/0001-3765201820170332.
69. Telascrea, M.; Araújo, C.C. de; Cavalheiro, A.J.; Marques, M.O.M.; Facanali, R.; Moraes, P.L.R. de Essential Oils from Leaves of *Cryptocarya* Spp from the Atlantic Rain Forest. *Quím. Nova* **2008**, *31*, 503–507, doi:10.1590/S0100-40422008000300007.
70. Brophy, J.J.; Forster, P.I.; Goldsack, R.J. Coconut Laurels: The Leaf Essential Oils from Four Endemic Australian *Cryptocarya* Species: *C. Bellendenkerana, C. Cocosoides, C. Cunninghamii* and *C. Lividula* (Lauraceae). *Natural Product Communications* **2016**, *11*, 1934578X1601100, doi:10.1177/1934578X1601100230.
71. Azhar, M.A.M.; Salleh, W.M.N.H.W.; Khamis, S. Essential Oil Composition of Three *Cryptocarya* Species from Malaysia. *Zeitschrift für Naturforschung C* **2020**, *75*, 297–301, doi:10.1515/znc-2020-0079.

72. Rali, T.; Wossa, S.W.; Leach, D.N. Comparative Chemical Analysis of the Essential Oil Constituents in the Bark, Heartwood and Fruits of *Cryptocarya Massoy* (Oken) Kosterm. (Lauraceae) from Papua New Guinea. *Molecules* **2007**, *12*, 149–154, doi:10.3390/12020149.
73. Telascrea, M.; de Araújo, C.C.; Marques, M.O.M.; Facanali, R.; de Moraes, P.L.R.; Cavalheiro, A.J. Essential Oil from Leaves of *Cryptocarya Mandioccana* Meisner (Lauraceae): Composition and Intraspecific Chemical Variability. *Biochemical Systematics and Ecology* **2007**, *35*, 222–232, doi:10.1016/j.bse.2006.09.015.
74. Setzer, W.N.; Haber, W.A. Leaf Essential Oil Composition of Five Species of *Beilschmiedia* from Monteverde, Costa Rica. *Natural Product Communications* **2007**, *2*, 1934578X0700200116, doi:10.1177/1934578X0700200116.
75. Salleh, W.M.N.H.W.; Ahmad, F.; Yen, K.H. Chemical Compositions and Biological Activities of the Essential Oils of *Beilschmiedia Madang* Blume (Lauraceae). *Arch. Pharm. Res.* **2015**, *38*, 485–493, doi:10.1007/s12272-014-0460-z.
76. Chaverri, C.; Cicció, J.F. Essential Oils From *Beilschmiedia Pendula* (Sw.) Hemsl. (Lauraceae) From Costa Rica. *J. Essent. Oil Res.* **2010**, *22*, 259–262, doi:10.1080/10412905.2010.9700320.
77. Yahyaa, M.; Matsuba, Y.; Brandt, W.; Doron-Faigenboim, A.; Bar, E.; McClain, A.; Davidovich-Rikanati, R.; Lewinsohn, E.; Pichersky, E.; Ibdah, M. Identification, Functional Characterization, and Evolution of Terpene Synthases from a Basal Dicot. *Plant Physiology* **2015**, *169*, 1683–1697, doi:10.1104/pp.15.00930.
78. Ma, Q.; Ma, R.; Su, P.; Jin, B.; Guo, J.; Tang, J.; Chen, T.; Zeng, W.; Lai, C.; Ling, F.; et al. Elucidation of the Essential Oil Biosynthetic Pathways in *Cinnamomum Burmannii* through Identification of Six Terpene Synthases. *Plant Science* **2022**, *317*, 111203, doi:10.1016/j.plantsci.2022.111203.
79. Chen, Y.; Li, Z.; Zhao, Y.; Gao, M.; Wang, J.; Liu, K.; Wang, X.; Wu, L.; Jiao, Y.; Xu, Z.; et al. The *Litsea* Genome and the Evolution of the Laurel Family. *Nat Commun* **2020**, *11*, 1675, doi:10.1038/s41467-020-15493-5.
80. Wang, X.; Xu, C.; Zheng, Y.; Wu, Y.; Zhang, Y.; Zhang, T.; Xiong, Z.Y.; Yang, H.-K.; Li, J.; Fu, C.; et al. Chromosome-Level Genome Assembly and Resequencing of Camphor Tree (*Cinnamomum Camphora*) Provides Insight into Phylogeny and Diversification of Terpenoid and Triglyceride Biosynthesis of *Cinnamomum*. *Horticulture Research* **2022**, *9*, uhac216, doi:10.1093/hr/uhac216.
81. Chang, Y.T.; Chu, F.H. Molecular Cloning and Characterization of Monoterpene Synthases from *Litsea Cubeba* (Lour.) Persoon. *Tree Genetics & Genomes* **2011**, *7*, 835–844, doi:10.1007/s11295-011-0377-3.
82. Lin, Y.L.; Lee, Y.R.; Huang, W.; Chang, S.T.; Chu, F.H. Characterization of S-(+)-Linalool Synthase from Several Provenances of *Cinnamomum Osmophloeum*. *Tree Genetics & Genomes* **2014**, *10*, 75–86, doi:10.1007/s11295-013-0665-1.
